# Pro‐Science Beliefs: The Role of Analytic Thinking and Epistemic Values

**DOI:** 10.1111/sjop.13114

**Published:** 2025-04-03

**Authors:** Sinem Yilmaz, Tomas Ståhl

**Affiliations:** ^1^ Department of Psychology University of Illinois at Chicago Chicago Illinois USA

**Keywords:** analytic thinking, importance of rationality, science beliefs, skepticism toward alternative medicine

## Abstract

The present research examined whether analytic thinking and valuing rationality predict pro‐science beliefs and skepticism toward alternative medicine. We hypothesized that analytic thinking would be more strongly positively associated with pro‐science beliefs and skepticism toward alternative medicine among people who strongly (vs. weakly) value being rational. In two studies, participants (*N*_S1_ = 470 and *N*_S2_ = 512) completed measures of analytic thinking, valuing rationality, pro‐science belief, and skepticism toward alternative medicine. We used hierarchical regression analyses to test our hypotheses. In Study 1, there was a stronger association between analytic thinking and both science beliefs and skepticism toward alternative medicine among those who strongly (vs. weakly) value being rational. In Study 2, the stronger association between analytic thinking and skepticism toward alternative medicine among those who strongly (vs. weakly) value being rational remained, but we did not replicate results from Study 1 on science beliefs. Pooled analyses across the two studies provided support for both of our hypotheses. Analytic thinking is particularly strongly associated with pro‐science beliefs and skepticism toward alternative medicine among people who value being rational. These findings highlight that both motivational and cognitive factors contribute to evidence‐based beliefs.

**Summary**

- In two studies, we examined whether analytic thinking and valuing rationality predict pro‐science beliefs and skepticism toward alternative medicine.
- Our results showed that analytic thinking is particularly strongly associated with pro‐science beliefs and skepticism toward alternative medicine among people who value being rational.
- These findings highlight that both motivational and cognitive factors predict evidence‐based beliefs.

Psychology has seen a surge in studies investigating why people adopt beliefs that do not cohere with established scientific evidence, such as paranormal (Lindeman and Svedholm‐Häkkinen 2016; Pennycook et al. 2012), supernatural (e.g., Darwin et al. 2011), and conspiracy beliefs (Swami et al. 2014) (i.e., epistemically suspect beliefs, Lobato et al. 2014). In parallel, other studies have examined what factors contribute to pro‐science beliefs: beliefs that are consistent with the best scientific evidence. One of the factors that has been shown to predict skepticism toward epistemically suspect beliefs and acceptance of pro‐science beliefs is analytic thinking.

According to dual process models of cognition, there are two different types of information processing: Type 1 processes, which are fast and frugal “intuitive” processes, and Type 2 processes, which are slow and deliberative processes that are effortful (e.g., De Neys 2012). Intuition, the output of Type 1 processes, can lead to errors, but it is important to note that they can also be adaptive and lead to accurate judgments in certain contexts (see Gigerenzer and Gaissmaier 2011). Analytic thinking arises from Type 2 processing and requires the ability to override intuitive responses. Importantly, there are individual differences in reliance on intuition versus analytic thinking, referred to as cognitive styles. An analytic cognitive style is distinct from a high need for cognition. Whereas the need for cognition reflects the general tendency to enjoy effortful thinking, analytic cognitive style refers to the ability and inclination to override intuitive responses when they conflict with logical reasoning (see Hill et al. 2013).

Importantly, analytic thinking has been found to be associated with disbelief in a variety of epistemically suspect beliefs, such as conspiracy theories (Swami et al. 2014) and paranormal beliefs (Pennycook et al. 2012). By contrast, people who are more inclined to think analytically are more likely to hold beliefs that are supported by scientific evidence, such as the notion that humans evolved through a process of natural selection (Gervais 2015) and that vaccination offers protection against infectious disease (Sarathchandra et al. 2018). There are several reasons why analytic thinking would promote epistemically rational beliefs. First, epistemically rational beliefs frequently compete with irrational beliefs that have more intuitive appeal (Norenzayan and Gervais 2013). Thus, people who rely on intuition rather than analytic thinking can be more inclined to trust their intuitions, whereas analytic thinkers are more likely to reflect on and override their initial irrational intuitions. Second, analytic thinkers (vs. intuitive thinkers) may have the skills required to detect the difference between strong and weak evidence, and thus be more skeptical of epistemically unfounded claims.

Despite analytic thinking's seemingly protective properties against epistemically suspect beliefs, other research has suggested that reflective thinking can enhance biased processing through motivated system 2 reasoning (Kahan et al. 2017). Indeed, Kahan et al. (2012) have found that the most (vs. least) reflective liberals are more inclined to believe the risks associated with climate change, but that the opposite pattern was obtained among conservatives. In other words, the more (vs. less) reflective conservatives were less likely to hold beliefs that are consistent with the scientific consensus on climate change (see also Drummond and Fischhoff 2017; Kahan 2013). A similar pattern has been observed with regard to other scientific issues, such as stem cell research, the big bang, and evolution (Bolsen et al. 2015; Drummond and Fischhoff 2017; Hamilton et al. 2012; Kahan et al. 2012). In all these cases, differences in scientifically plausible beliefs have been the largest among the most reflective opposing partisans. This pattern of results suggests that, under certain circumstances, reflective thinkers may be better equipped to use their reasoning skills and/or domain knowledge to align their evaluation of evidence with their political identity.

Alternatively, Pennycook et al. (2020) has argued that politically motivated reasoning may not be directly responsible for the observation that partisan reasoning biases are accentuated among more (vs. less) analytic thinkers. Rather, Pennycook suggests that *prior beliefs* that are associated with one's political identity are likely to be more developed and detailed among analytic (vs. intuitive) thinkers, and this difference could be the factor explaining this effect (Tappin et al. 2020a, 2020b, 2021)^1^. Specifically, because highly analytic partisans are more likely to have more developed and diverging prior beliefs, they should be particularly inclined to respond differently to new information and, hence, end up with more diverging conclusions than less analytic thinkers. Thus, whereas the ideology/identity account suggests that motivated reasoning is very much a hot (motivated) cognition phenomenon fueled by identity (or other motivational/value driven) concerns, the prior beliefs account views this reasoning bias primarily as a cold (not motivated) cognitive phenomenon. Despite their differences in their explanation for biases in reasoning (ideology and political identities vs. prior beliefs associated with ideologies), they both argue that ideological positions can prevent us from forming beliefs that are consistent with the best available evidence. In either case, cognitive sophistication might magnify biased reasoning either because new information is inconsistent with the person's specific prior beliefs or their political identities. Notably, however, more recent studies have failed to replicate this effect, and there is a growing consensus that analytic thinking typically improves reasoning about epistemically suspect beliefs and pro‐science beliefs, or at the very least does not accentuate bias (see Baker et al. 2020; Persson et al. 2021; Stagnaro et al. 2023).

A possible way to further reduce the influence of ideology/identity or prior beliefs when evaluating new evidence would be to have the pursuit of truth as a highly salient goal. The goal to pursue the truth could help people evaluate the quality of arguments and evidence in a more objective fashion, rather than favoring information that is consistent with one's identity, ideology, or prior beliefs (Ståhl and Cusimano 2024). Supporting this argument, valuing one's own beliefs being based on logic and evidence has been found to be positively associated with belief in science and negatively associated with belief in paranormal phenomena and conspiracy theories (Ståhl and van Prooijen 2018). In a similar vein, Pennycook et al. (2020) found that believing that beliefs should change according to evidence was positively associated with science beliefs. Notably, this association was stronger for liberals than conservatives.

It is also possible that having an open mind about evidence may only be a part of the equation. Consistent with research on attitude change (Petty et al. 1986), people may need sufficient ability and motivation to be rational to adopt scientifically plausible beliefs. Indeed, the value given to being rational has been found to moderate the relationship between analytic thinking and unfounded beliefs, such as conspiracy theories and paranormal phenomena (Ståhl and van Prooijen 2018). Specifically, among those who strongly value rationality, analytic thinking was associated with a lower inclination to believe such epistemically suspect beliefs, while this relationship was weaker among people who valued rationality less. Notably, similar findings have been obtained when the motivation to be rational (see Adam‐Troian et al. 2019) and the ability to engage in analytic thinking was manipulated (Ståhl et al. 2024). In short, there is growing evidence that skepticism toward alternative medicine is particularly likely to be promoted by analytic thinking when people are motivated to be rational.

## 1 The Present Research

The present research aims to examine how analytic thinking and valuing rationality promote endorsement of pro‐science beliefs. Previous studies have looked at either the impact of motivation to be rational or analytic thinking on pro‐science beliefs but not their interaction. Although Ståhl and van Prooijen (2018) showed the influence of valuing rationality and analytic thinking on skepticism toward various epistemically suspect beliefs, their studies did not examine their associations with scientifically *supported* beliefs. Thus, it is unclear whether the combination of analytic thinking and valuing rationality promotes truth discernment or merely increases skepticism. Consequently, finding the reversed effect on pro‐science beliefs (i.e., that the combination of motivation to be rational and analytic thinking promote science beliefs) would indicate that these factors promote discernment of what is true from what is false. We address this gap in the literature directly in two studies.

## 2 Study 1

The pre‐registration (https://aspredicted.org/ZR9_C76) and all research materials and data are available (https://osf.io/mc2hs/?view_only=f62f091b4f8e44d2be1a0b2287b8118e). We anticipated analytic thinking to be more positively associated with pro‐science beliefs among people who value being rational strongly (rather than weakly).

### 2.1 Method

#### 2.1.1 Participants

Participants who lived in the United States were recruited from Amazon Mechanical Turk. We conducted a power analysis in GPower to estimate our sample size. We determined the required sample size to be at least 387 to detect an effect size *f* ^2^ = 0.02 and power = 0.80 in linear multiple regression tests with three predictors (analytic thinking, the importance given to rationality, and their interaction). We decided on 500 participants as an appropriate sample size to compensate for potential subject dropout and exclusions. Twenty participants who did not answer all the questions were excluded. Consistent with our pre‐registration, 10 participants who failed both of our attention check questions, or our bot check question were excluded. The resulting final sample (*N* = 470, Mean_age_ = 41.49, SD_age_ = 12.0) consisted of 234 males and 234 females (2 participants did not report their gender). The majority of the participants had bachelor's or higher degrees in educational level; 192 participants (40.9%) had bachelor's, 58 (12.3%) had master's, 6 (1.3%) had professional degrees, and 7 (1.5%) had doctoral degrees 1 participant had less than high school (0.2%), 55 participants had a high school degree (11.7%), 93 participants had college without a degree (19.8%), and 58 were associates (12.3%).

#### 2.1.2 Materials

##### 2.1.2.1 Importance of Rationality Scale (IRS; Ståhl et al. 2016)

The six‐item IRS measures the extent to which people value forming their beliefs based on evidence and logic. Items were rated on a scale from 1 (strongly disagree) to 7 (strongly agree), with higher scores representing a stronger value of being epistemically rational. The six items were averaged together to create a measure of personal importance attached to being rational (*⍺* = 0.85).

##### 2.1.2.2 Analytic Cognitive Style (AT)

Participants completed the three‐item Cognitive Reflection Test (CRT; Frederick 2005) and the 4‐item CRT 2 (Thomson and Oppenheimer 2016). These tests contain a total of seven problems that cue intuitive but wrong answers. Suppressing the intuitive answers and giving the correct answer is considered to be an indicator of analytic thinking. Questions were presented in an open‐ended format with correct responses coded as 1. The seven items were averaged together to create a reliable analytic thinking scale (AT) on which higher scores represent greater analytic thinking (*⍺* = 0.78).

##### 2.1.2.3 Pro‐Science Beliefs (Pennycook et al. 2023)

The pro‐science beliefs items measure participants' agreement with 17 statements about various scientific and pseudoscientific issues on a scale of 1 (strongly disagree) to 6 (strongly agree). As stated in our pre‐registration, we intended to divide the pro‐science beliefs items into a liberal and a conservative pro‐science subscale, as outlined in the original work by Pennycook et al. (2023). In that original article, the subsets of items were categorized a priori as conservative or liberal pro‐science beliefs based on the political context in which they were developed. Specifically, the four liberal pro‐science items included belief in global warming, evolution, the Big Bang, and sex ambiguity. Conservative pro‐science items comprised the remaining 13 items, such as nuclear power safety, IQ heritability, testosterone and athleticism, and stereotype accuracy. This subset was intended to capture issues on which conservatives were more inclined to hold a pro‐scientific stance. However, for reasons laid out in the section ‘Deviations from Pre‐registration’ below, we ultimately opted to deviate from this preregistered plan.

##### 2.1.2.4 Political Ideology

Participants were asked to indicate their stance on social, economic, and general issues separately on scales from 1 (very liberal) to 7 (very conservative). All three items were averaged together to create one political ideology score (*⍺* = 0.96).

##### 2.1.2.5 Additional Measures

For exploratory purposes, the Numeracy test (Schwartz et al. 1997), the Wordsum test (Huang and Hauser 1998), the Moralized Rationality Scale (MRS; Ståhl et al. 2016), the Actively Open‐Minded Thinking Scale About Evidence (AOT‐E; Pennycook et al. 2020), and a single‐item measuring trust in scientists were also presented to the participants. The Numeracy and Wordsum tests were averaged to measure cognitive ability. Participants were also asked a set of demographic questions, including gender, age, level of education, political orientation, and religiosity.

#### 2.1.3 Procedure

Following the informed consent, participants were presented with the following measurements: Pro‐Science Beliefs items (Pennycook et al. 2023), analytic thinking measures (CRT 1–2), and the importance given to being rational (IRS)^2^. Two attention check questions were embedded in the Pro‐Science Belief items and the IRS.

#### 2.1.4 Deviations From Pre‐Registration

We observed notable inconsistencies in the correlations between Pro‐Science belief items and political ideology. Specifically, items such as IQ heritability, gender transition, testosterone, wage gap sexism, and SAT correlated positively with conservatism, which is consistent with the original study (Pennycook et al. 2023). However, items like GMO safety, acupuncture rejection, vaccine safety, homeopathy rejection, essential oils rejection, detox rejection, and stereotype accuracy correlated positively with liberalism, contrary to the original findings.

We also conducted a confirmatory factor analysis to test the original two‐factor model with items expected to load on either the liberal or conservative science beliefs factor. The results showed that this model did not fit the data well, *χ* ^2^(118) = 737.23, *p* < 0.001, RMSEA = 0.106, CFI = 0.727, AIC = 26944.27, TLI = 0.685, BIC = 27089.61. The two factors were negatively correlated (*r* = −0.45, *p* < 0.001).

In light of these findings, we decided to abandon the pre‐registered plan to group the items based on their expected relationships with political ideology. Instead, we conducted an exploratory factor analysis (EFA) and a confirmatory factor analysis (CFA) on the data from Study 1, and followed up with another CFA on the data from Study 2. Based on these analyses, we ended up creating two alternative subscales capturing science beliefs and skepticism toward alternative medicine, respectively. The details and results of these factor analyses are reported in the Supporting Information S1.

#### 2.1.5 Results and Discussion

All analyses were completed with R (R Core Team 2024). Table 1 presents the means, standard deviations, and zero‐order correlations. Our hypotheses were tested using hierarchical regression analyses. In the first step, the value given to being rational and analytic thinking (both predictors were standardized) were entered into the model. In the second step, we entered the interaction term.

**TABLE 1:** Means, standard deviations, and correlations with 95% confidence intervals (Study 1).

Variable	*M*	SD	1	2	3	4	5	6	7	8	9
1. AT	4.55	2.01									
2. IRS	5.74	0.92	0.18**								
			[0.09, 0.26]								
3. MRS	4.00	1.23	−0.03	0.38**							
			[−0.12, 0.06]	[0.30, 0.46]							
4. Combined PSB	4.07	0.61	0.31**	0.30**	0.01						
			[0.23, 0.39]	[0.22, 0.38]	[−0.08, 0.10]						
5. Science Beliefs	4.43	1.19	0.14**	0.33**	0.17**	0.62**					
			[0.05, 0.23]	[0.25, 0.41]	[0.08, 0.26]	[0.56, 0.67]					
6. Skepticism of Alternative Medicine	3.84	1.12	0.30**	0.17**	−0.01	0.84**	0.39**				
			[0.21, 0.38]	[0.08, 0.25]	[−0.10, 0.08]	[0.81, 0.87]	[0.31, 0.46]				
7. Political Ideology	3.47	1.75	−0.07	−0.20**	−0.09	−0.36**	−0.65**	−0.34**			
			[−0.16, 0.02]	[−0.28, −0.11]	[−0.18, 0.00]	[−0.43, −0.27]	[−0.70, −0.60]	[−0.41, −0.25]			
8. Gender [baseline as male]	1.51	0.51	−0.12*	−0.09*	−0.06	−0.22**	0.04	−0.22**	−0.14**		
			[−0.20, −0.03]	[−0.18, −0.00]	[−0.15, 0.03]	[−0.30, −0.13]	[−0.05, 0.13]	[−0.30, −0.13]	[−0.23, −0.05]		
9. Education	4.31	1.36	0.13**	0.07	0.10*	0.10*	0.07	0.03	0.04	−0.06	
			[0.04, 0.22]	[−0.02, 0.16]	[0.01, 0.19]	[0.01, 0.19]	[−0.02, 0.16]	[−0.06, 0.12]	[−0.05, 0.13]	[−0.15, 0.03]	
10. Age	41.49	11.99	0.06	0.03	−0.07	−0.09	−0.01	−0.17**	0.13**	0.05	0.06
			[−0.03, 0.15]	[−0.06, 0.13]	[−0.16, 0.02]	[−0.18, 0.00]	[−0.11, 0.08]	[−0.26, −0.08]	[0.04, 0.22]	[−0.04, 0.14]	[−0.03, 0.15]

*Note:* Combined PSB = Original 17 items Pro‐Science Beliefs Scale.

Abbreviations: AOT‐E = actively open‐minded thinking about evidence, AT = analytic thinking, IRS = Importance of Rationality Scale, MRS = Moralized Rationality Scale.

* *p* < 0.05.

** *p* < 0.01.

Because both science beliefs and skepticism toward alternative medicine were related to being liberal (see Table 1), we also conducted separate hierarchical regression analyses in which we controlled for political ideology and its interactions with analytic thinking and the value given to being rational (see Supporting Information S1 for these regression tables).

##### 2.1.5.1 Science Beliefs

**FIGURE 1:**
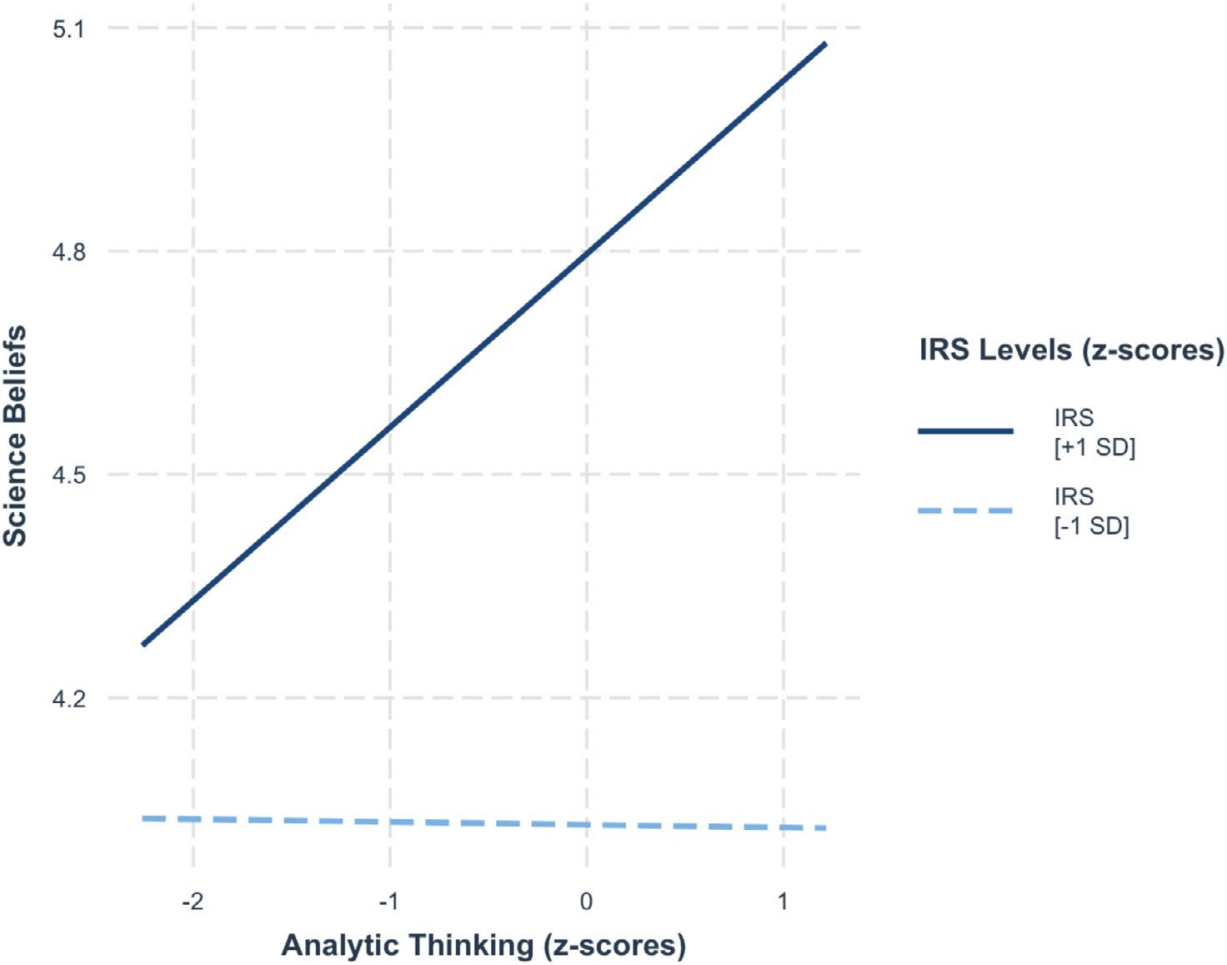
Predicted science belief scores as a function of analytic thinking (AT) and the importance of rationality (IRS) (Study 1).

Step 1 explained significant variance in science beliefs,^3^ *F*(2, 467) = 31.1, *p* < 0.001, *R* ^2^ = 0.118. Consistent with previous studies, both analytic thinking and the value given to being rational were positively associated with science beliefs, *b* = 0.10, SE = 0.05, *t* = 2.03, *p* = 0.042, and *b* = 0.38, SE = 0.05, *t* = 7.14, *p* < 0.001, respectively. Notably, a significant amount of additional variance was explained in Step 2, *F*(1, 466) = 6.14, *p* = 0.021, ∆*R* ^2^ = 0.007. Consistent with our hypothesis, the value given to being rational moderated the relationship between analytic thinking and science beliefs, *b* = 0.11, SE = 0.05, *t* = 2.32, *p* = 0.021. As seen in Figure 1, simple slope analysis showed that, consistent with our hypothesis, analytic thinking was positively associated with science beliefs among people who valued being rational strongly (+1 SD), *b* = 0.23, SE = 0.08, *t* = 3.02, *p* < 0.001, but not among those who valued being rational weakly (−1 SD), *b* = 0.01, SE = 0.07, *t* = 0.05, *p* = 0.96.

**FIGURE 2:**
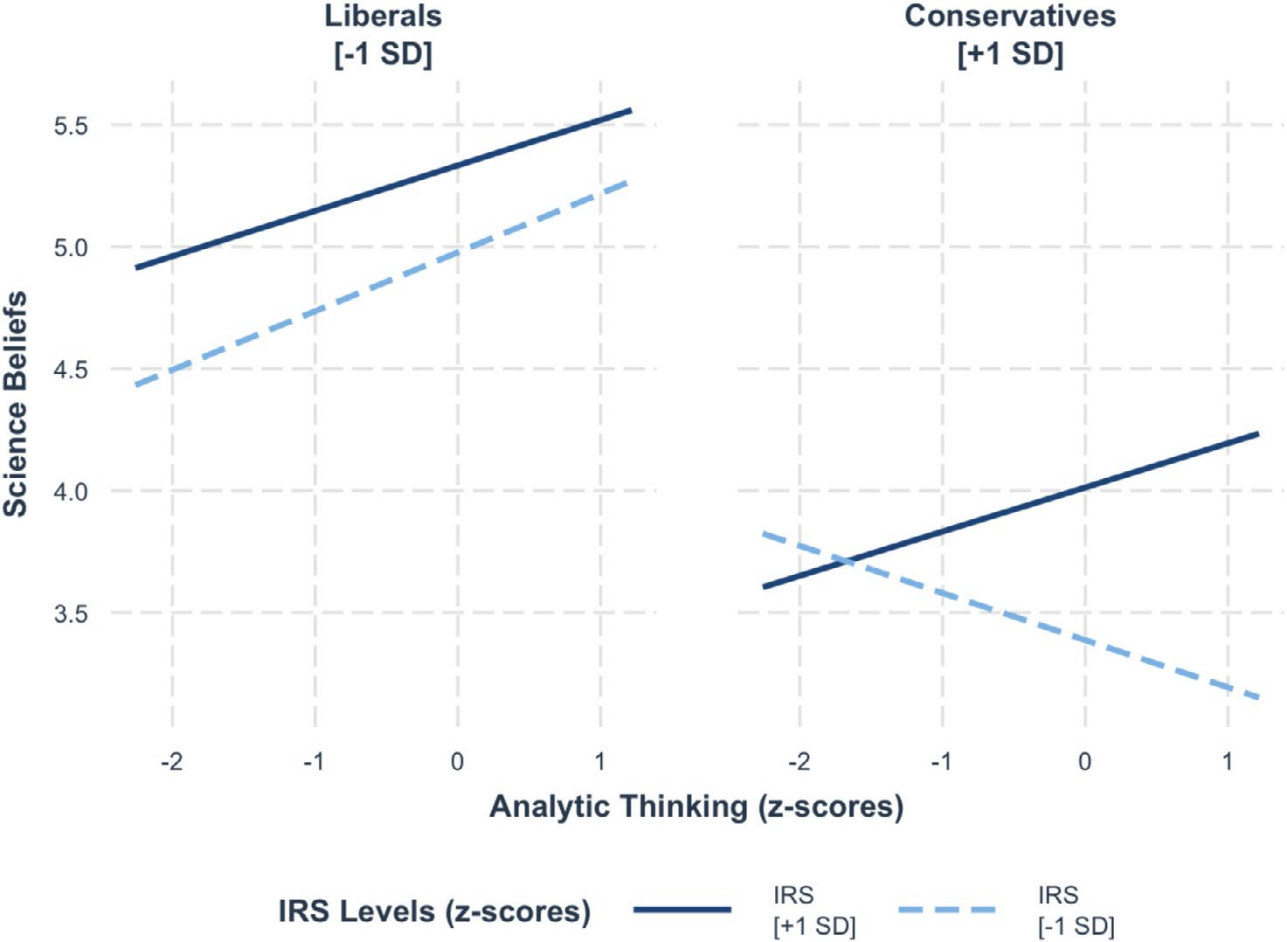
Predicted science belief scores as a function of analytic thinking (AT), the importance of rationality (IRS), and political ideology (Study 1).

A follow‐up analysis demonstrated that our hypothesized effect on science beliefs was qualified by political ideology, *b* = 0.10, SE = 0.04, *t* = 2.71, *p* = 0.01. Analytic thinking interacted with the value given to being rational among conservatives (+1 SD), *F*(1, 462) = 10.59, *p* = 0.001, but this interaction was not significant among liberals (−1 SD), *F*(1, 462) = 0.1, *p* = 0.75. As seen in Figure 2, simple slope analyses showed that among conservatives who valued being rational weakly (−1 SD), analytic thinking was negatively associated with science beliefs, *b* = −0.19, SE = 0.07, *t* = −2.65, *p* = 0.01, whereas analytic thinking was positively associated with science beliefs among conservatives who valued being rational strongly (+ 1 SD), *b* = 0.18, SE = 0.09, *t* = 2.06, *p* = 0.04.

##### 2.1.5.2 Skepticism Toward Alternative Medicine

**FIGURE 3:**
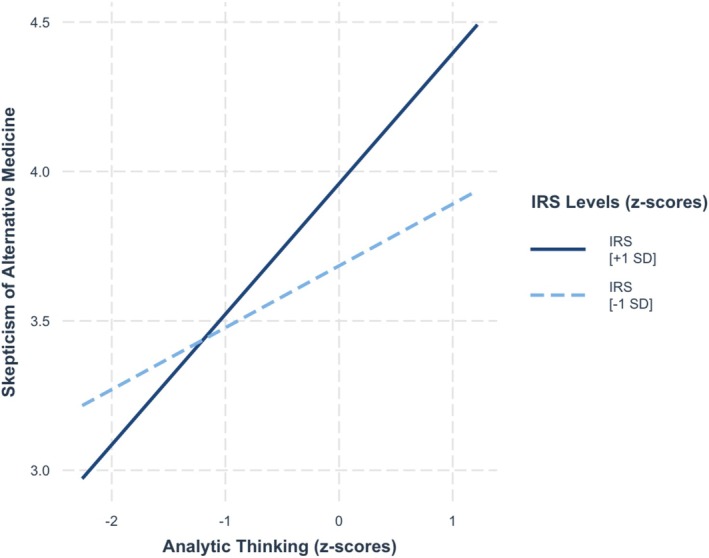
Predicted skepticism of alternative medicine scores as a function of analytic thinking (AT) and the importance of rationality (IRS) (Study 1).

Step 1 explained a significant amount of variance in skepticism of alternative medicine,^4^ *F*(2, 467) = 29.0, *p* < 0.001, *R* ^2^ = 0.10. Consistent with previous studies, both analytic thinking and the value given to being rational were positively associated with skepticism of alternative medicine, *b* = 0.31, SE = 0.04, *t* = 6.75, *p* < 0.001, and *b* = 0.12, SE = 0.04, *t* = 2.77, *p* = 0.006, respectively. Notably, a significant amount of additional variance was explained in Step 2, *F*(1, 466) = 4.26, *p* = 0.02, ∆*R* ^2^ = 0.01, as the value given to being rational moderated the relationship between analytic thinking and skepticism of alternative medicine, *b* = 0.11, SE = 0.05, *t* = 2.38, *p* = 0.02. As seen in Figure 3, simple slope analysis showed that analytic thinking was more strongly positively associated with skepticism of alternative medicine among people who valued being rational strongly (+ 1 SD), *b* = 0.40, SE = 0.07, *t* = 6.04, *p* < 0.001, than among those who valued weakly (−1 SD), *b* = 0.22, SE = 0.06, *t* = 3.55, *p* = < 0.001.

Moreover, there was no three‐way interaction involving political ideology, *b* = 0.06, SE = 0.05, *t* = 1.47, *p* = 0.14. When controlling for political ideology, this two‐way interaction remained significant, *b* = 0.10, SE = 0.04, *t* = 2.26, *p* = 0.03.

In conclusion, Study 1 provided support for our hypotheses. Specifically, results indicated that analytic thinking was more strongly associated with science beliefs, as well as skepticism toward alternative medicine, among those who value being rational strongly (rather than weakly). Notably, political ideology influenced this hypothesized effect on science beliefs, such that it was only significant among conservatives.

A limitation of this study was the fact that participants affiliated with the Democratic Party were overrepresented (*n* = 232), compared to participants associated with the Republican Party (*n* = 108). This skewed distribution may have introduced bias and potential confounding factors into our analysis. Recognizing this potential limitation, we attempted to address this issue in Study 2 by recruiting a larger sample with a more balanced representation of conservatives and liberals, thereby enabling a comprehensive examination of the phenomenon under investigation.

## 3 Study 2

In study 2, we aimed to replicate our findings with a more balanced sample. To do that, we set a quota for the number of democrats and republicans to recruit an equal number of democrats and republicans. This study was not pre‐registered, but it was identical to Study 1 except for the mentioned differences.

### 3.1 Method

#### 3.1.1 Participants

Participants who lived in the United States were recruited from Amazon Mechanical Turk. We conducted a power analysis in GPower to estimate our sample size. We determined the required sample size to be at least 387 to detect an effect size *f* ^2^ = 0.02 and power = 0.80 in linear multiple regression tests with three predictors (analytic thinking, the importance given to rationality, and their interaction). To compensate for potential subject dropout and exclusions, we decided on 550 participants as an appropriate sample size. Twenty‐seven participants who did not answer all of the questions and 11 participants who failed both our attention check questions and our bot check question were excluded. The resulting sample (*N* = 512, Mean_age_ = 39.9, SD_age_ = 11.4) consisted of 297 males, 209 females, and 6 non‐binary participants. The majority of the participants had bachelor's or higher degrees in educational level; 230 participants (44.9%) had bachelor's, 68 (13.3%) had master's, 3 (0.6%) had professional degrees, and 12 (2.3%) had doctoral degrees. Two participants had less than a high school (0.4%), 55 participants had a high school degree (10.7%), 89 participants had a college without a degree (17.4%), and 53 were associates (10.4%).

#### 3.1.2 Materials

Materials were virtually identical to the ones used in Study 1. The only changes made were removing the measurements included in Study 1 for exploratory purposes.

#### 3.1.3 Procedure

Upon completing the informed consent, participants were presented with the questionnaire. Unlike in Study 1, the order of the instruments was the same for all participants with the pro‐science beliefs items presented first (science beliefs, ⍺ = 0.78 and skepticism of alternative medicine, ⍺ =0.85).

#### 3.1.4 Results and Discussion

Table 2 presents the means, standard deviations, and zero‐order correlations. As in Study 1, we used hierarchical multiple regression analyses to test our hypotheses. In the first step, we entered the value given to being rational and analytic thinking. In the second step, we entered the interaction term.

**TABLE 2:** Means, standard deviations, and correlations with 95% confidence intervals (Study 2).

Variable	*M*	SD	1	2	3	4	5	6	7	8	9
1. AT	4.54	2.04									
2. IRS	5.80	0.90	0.21**								
			[0.13, 0.30]								
3. MRS	3.95	1.27	−0.12**	0.28**							
			[−0.21, −0.04]	[0.20, 0.36]							
4. Combined PSB	4.01	0.68	0.45**	0.37**	0.07						
			[0.37, 0.51]	[0.30, 0.45]	[−0.02, 0.16]						
5. Science Beliefs	4.18	1.32	0.15**	0.35**	0.24**	0.67**					
			[0.07, 0.23]	[0.27, 0.42]	[0.16, 0.32]	[0.62, 0.72]					
6. Skepticism of Alternative Medicine	3.74	1.18	0.46**	0.24**	0.04	0.86**	0.45**				
			[0.39, 0.53]	[0.16, 0.32]	[−0.05, 0.12]	[0.84, 0.88]	[0.38, 0.52]				
7. Political Ideology	4.00	1.87	−0.17**	−0.20**	−0.17**	−0.46**	−0.65**	−0.44**			
			[−0.25, −0.08]	[−0.28, −0.12]	[−0.25, −0.08]	[−0.52, −0.38]	[−0.70, −0.60]	[−0.50, −0.36]			
8. Gender [baseline as male]	1.43	0.52	−0.07	−0.01	−0.13**	−0.13**	−0.04	−0.11*	−0.04		
			[−0.15, 0.02]	[−0.09, 0.08]	[−0.22, −0.05]	[−0.22, −0.05]	[−0.13, 0.04]	[−0.19, −0.02]	[−0.13, 0.04]		
9. Education	4.43	1.37	0.05	0.01	0.11*	0.06	0.09*	0.02	−0.06	0.02	
			[−0.03, 0.14]	[−0.08, 0.09]	[0.02, 0.19]	[−0.03, 0.15]	[0.01, 0.18]	[−0.07, 0.10]	[−0.15, 0.03]	[−0.07, 0.11]	
10. Age	39.88	11.43	0.10*	0.12**	−0.15**	−0.02	−0.09*	−0.08	0.23**	0.15**	0.07
			[0.01, 0.18]	[0.04, 0.21]	[−0.23, −0.06]	[−0.11, 0.07]	[−0.17, −0.00]	[−0.16, 0.01]	[0.15, 0.31]	[0.06, 0.23]	[−0.02, 0.16]

*Note:* Combined Pro‐SB = Original 17 items Pro‐Science Beliefs Scale.

Abbreviations: AT = analytic thinking, IRS = Importance of Rationality Scale, MRS = Moralized Rationality Scale.

* *p* < 0.05.

** *p* < 0.01.

As in Study 1, because both science beliefs and skepticism toward alternative medicine were related to being liberal (see Table 2), we also conducted separate hierarchical regression analyses in which we controlled for political ideology and its interactions with the value given to being rational and analytic thinking (see Supporting Information S1 for these regression tables).

##### 3.1.4.1 Science Beliefs

**FIGURE 4:**
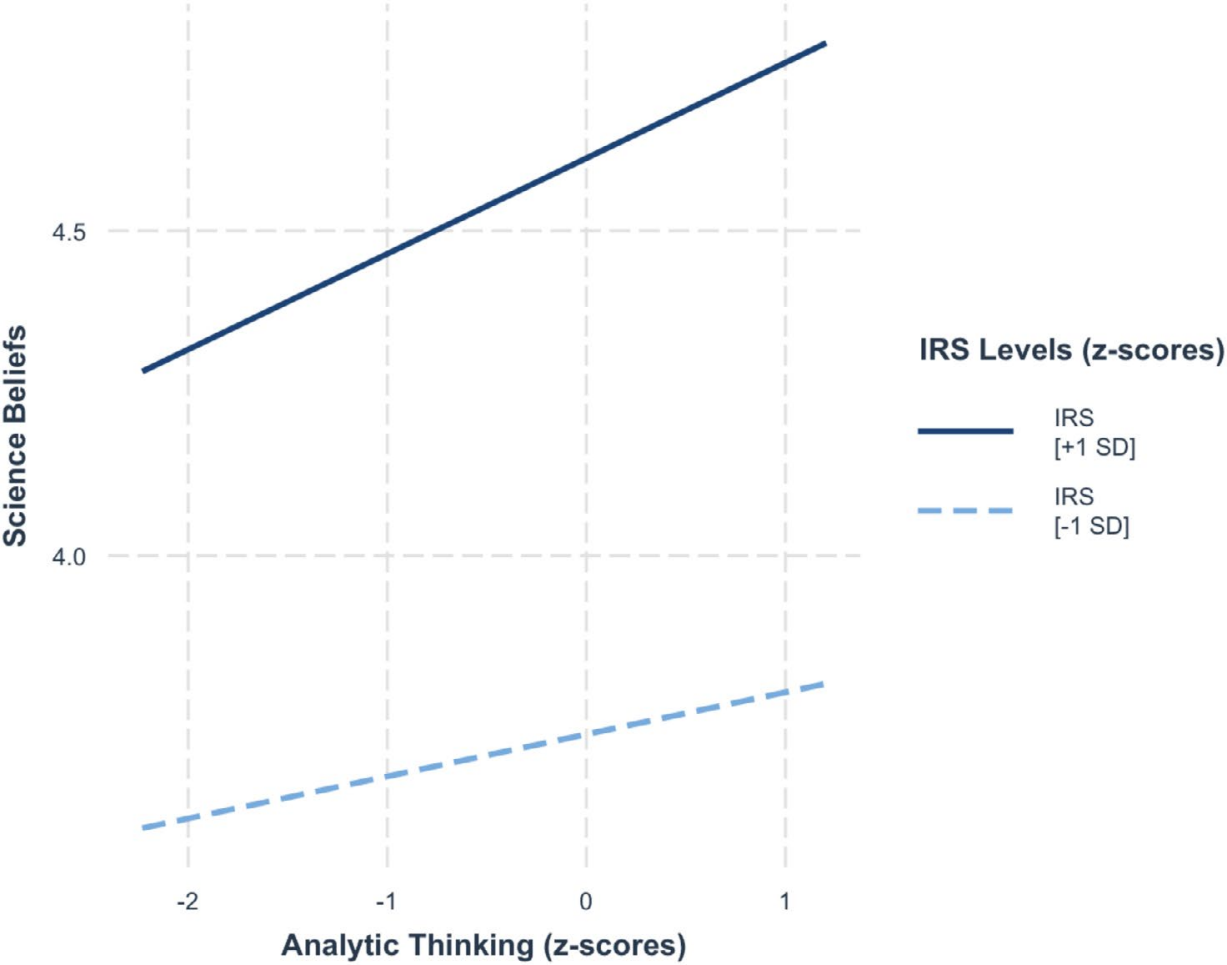
Predicted science beliefs scores as a function of analytic thinking (AT) and the importance of rationality (IRS) (Study 2).

Step 1 explained significant variance in science beliefs, *F*(2, 509) = 38.4, *p* < 0.001, *R* ^2^ = 0.131. As seen in Figure 4, both the values given to being rational and analytic thinking were positively associated with science beliefs, *b* = 0.44, SE = 0.05, *t* = 7.85, *p* < 0.001, and *b* = 0.11, SE = 0.05, *t* = 1.90, *p* = 0.058, respectively. Unlike in Study 1, a significant amount of additional variance was not explained in Step 2, *F*(1, 542) = 0.57, *p* = 0.45, ∆*R* ^2^ = 0.002, as the value given to being rational did not moderate the relationship between analytic thinking and science beliefs, *b* = 0.04, SE = 0.05, *t* = 0.75, *p* = 0.45. Thus, no support was found for our hypothesis.

**FIGURE 5:**
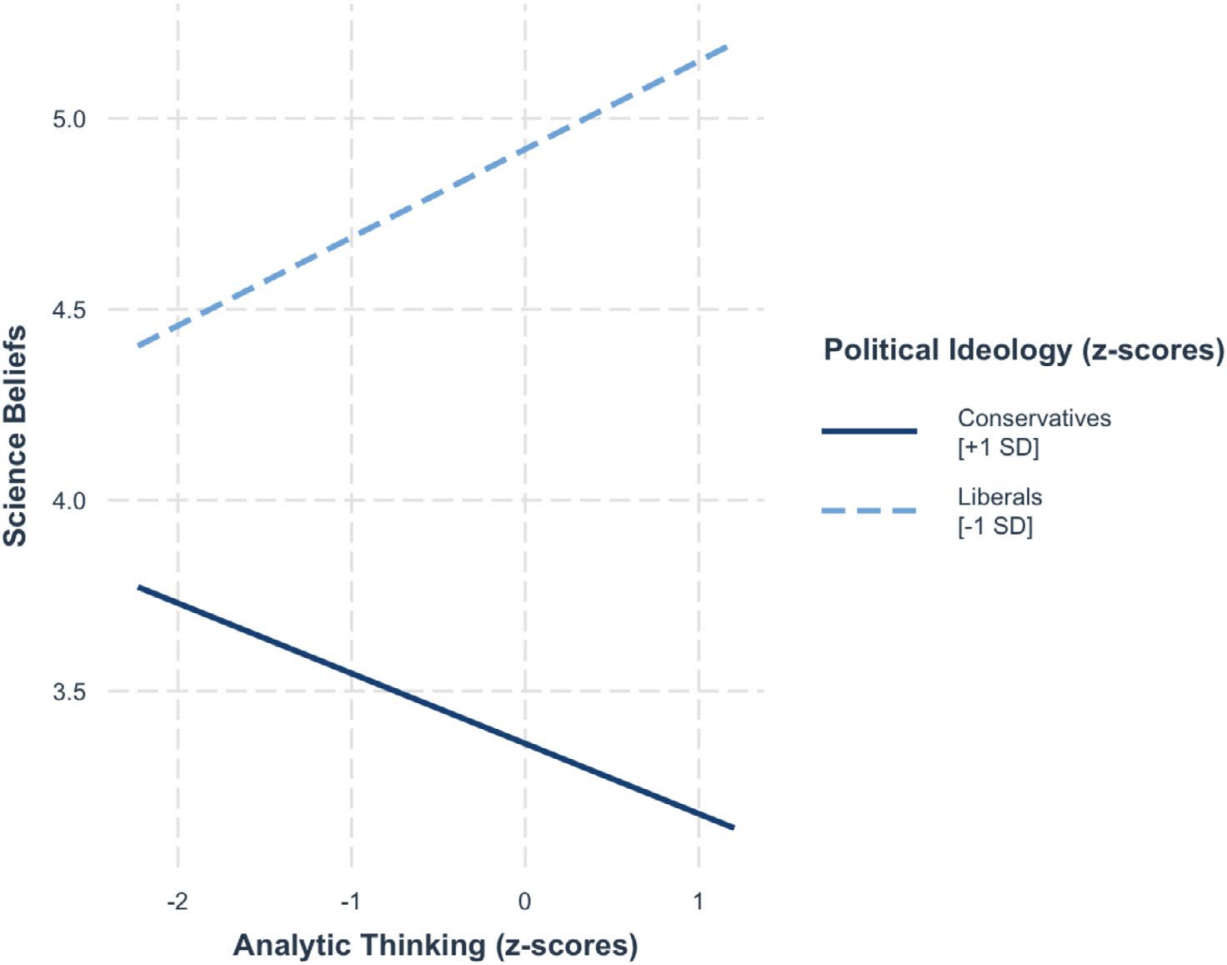
Predicted science beliefs scores as a function of analytic thinking (AT) and political ideology (Study 2).

Moreover, unlike in Study 1, there was no three‐way interaction involving political ideology, *b* = −0.007, SE = 0.02, *t* = −0.28, *p* = 0.77. However, there was a two‐way interaction between analytic thinking and political ideology, *b* = −0.21, SE = 0.02, *t* = −4.57, *p* < 0.001. As seen in Figure 5, simple slope analysis showed that analytic thinking was positively associated with science beliefs among liberals, *b* = 0.24, SE = 0.07, *t* = 3.56, *p* < 0.001, and negatively associated among conservatives, *b* = −0.18, SE = 0.06, *t* = −3.12, *p* < 0.001. This finding is consistent with the idea that either analytic thinking facilitates motivated reasoning or the influence of more established prior beliefs.

##### 3.1.4.2 Skepticism Toward Alternative Medicine

**FIGURE 6:**
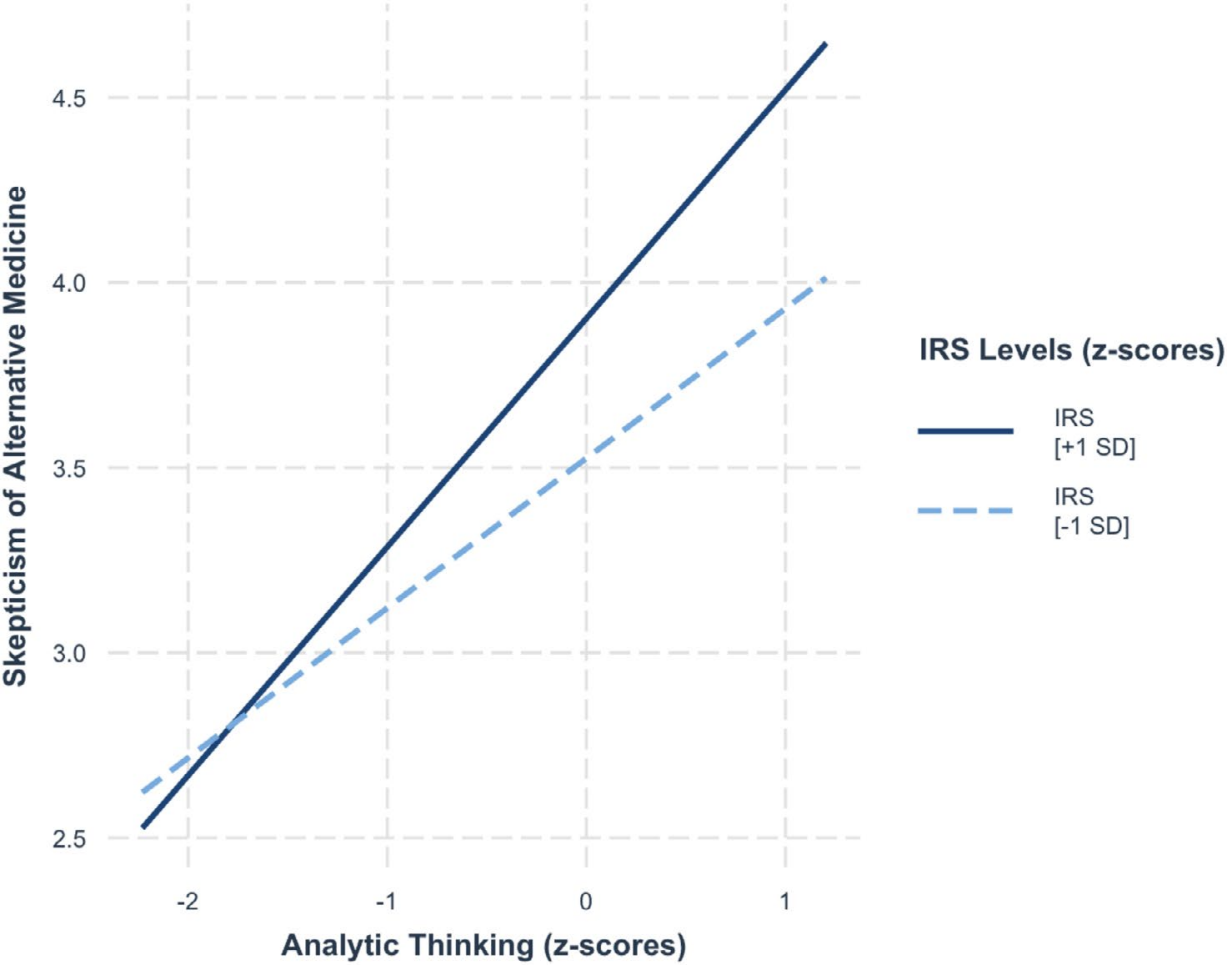
Predicted skepticism of alternative medicine scores as a function of analytic thinking (AT) and the importance of rationality (IRS) (Study 2).

Step 1 explained a significant amount of variance in skepticism of alternative medicine, *F*(2, 509) = 86.96, *p* < 0.001, *R* ^2^ = 0.25. Consistent with previous studies, both analytic thinking and the value given to being rational were positively associated with skepticism of alternative medicine, *b* = 0.53, SE = 0.04, *t* = 11.55, *p* < 0.001 and *b* = 0.18, SE = 0.04, *t* = 3.79, *p* < 0.001, respectively. Notably, a significant amount of additional variance was explained in Step 2, *F*(1, 508) = 4.61, *p* = 0.021, ∆*R* ^2^ = 0.01. Consistent with our hypothesis, the value given to being rational moderated the relationship between analytic thinking and skepticism of alternative medicine, *b* = 0.10, SE = 0.04, *t* = 2.30, *p* = 0.021^5^. Our follow‐up simple slope analyses showed that, as seen in Figure 6, analytic thinking was more strongly positively associated with skepticism of alternative medicine among people who valued being rational strongly (+ 1 SD), *b* = 0.63, SE = 0.06, *t* = 9.74, *p* < 0.001, than among people who valued being rational weakly (− 1 SD), *b* = 0.43, SE = 0.07, *t* = 6.69, *p* < 0.001. Political ideology did not qualify these findings, as there was no three‐way interaction, *b* = −0.02, SE = 0.043, *t* = −0.53, *p* = 0.66.

In summary, we once again received some support for our hypothesis. However, unlike in Study 1, we now found support for our prediction regarding skepticism toward alternative medicine, but not regarding science beliefs. Specifically, analytic thinking was more strongly associated with skepticism toward alternative medicine among those who value being rational strongly (rather than weakly).

## 4 Studies 1–2 Combined

Due to the somewhat inconsistent findings across studies, we decided to pool the two datasets together to see which relationships held up.

### 4.1 Science Beliefs

**FIGURE 7:**
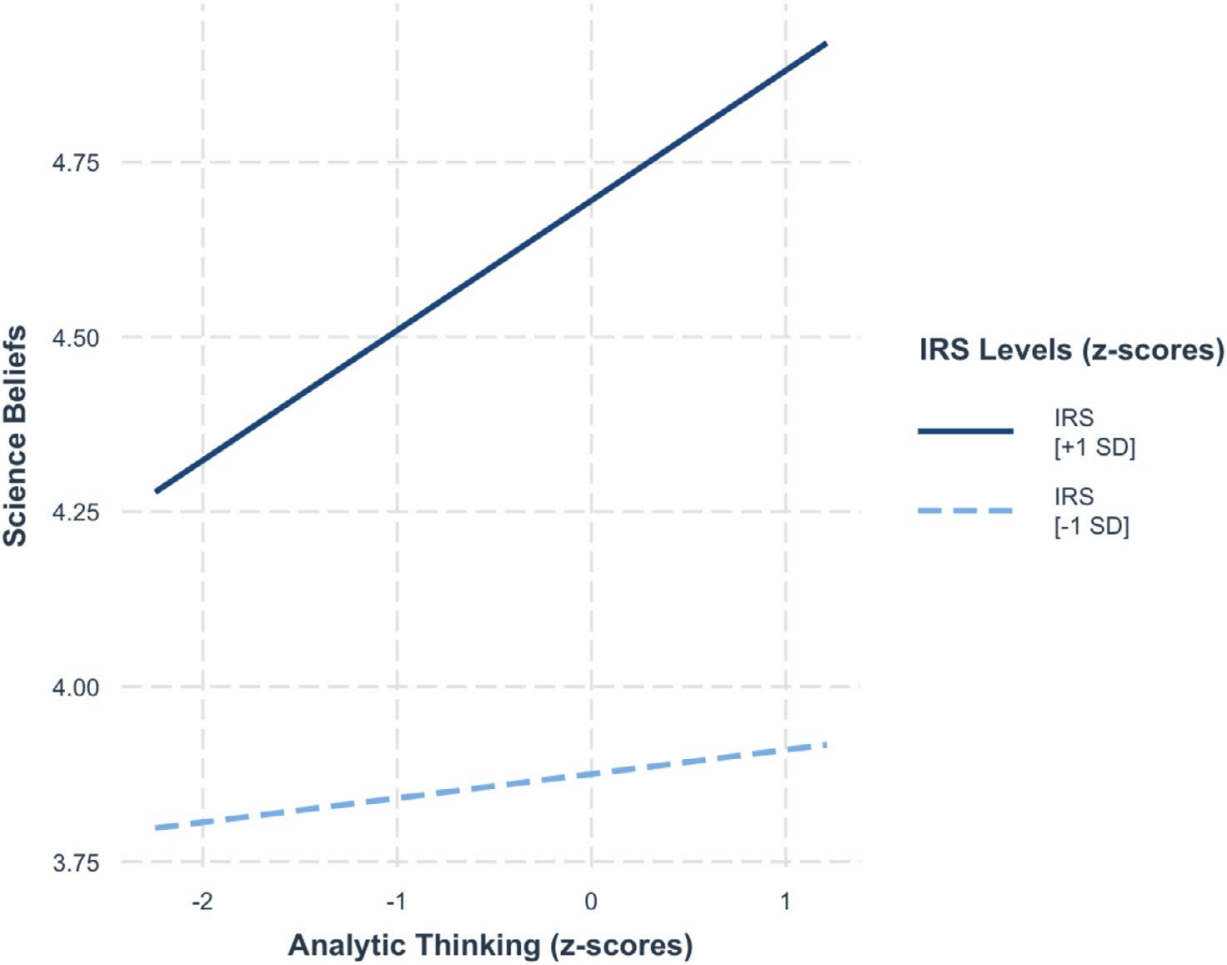
Predicted science beliefs scores as a function of analytic thinking (AT) and the importance of rationality (IRS) (Studies 1 and 2 Combined).

Step 1 explained a substantial amount of variance in science beliefs, *F*(2, 979) = 67.7, *p* < 0.001, *R* ^2^ = 0.122. Consistent with previous studies, both analytic thinking and the value given to being rational were positively associated with science beliefs, *b* = 0.12, SE = 0.038, *t* = 3.1, *p* = 0.002 and *b* = 0.40, SE = 0.038, *t* = 10.4, *p* < 0.001, respectively. Importantly, Step 2 explained significant additional variance, *F*(1, 978) = 4.92, *p* = 0.045, ∆*R* ^2^ = 0.004. Consistent with our hypothesis (and Study 1), the value given to being rational moderated the relationship between analytic thinking and science beliefs, *b* = 0.08, SE = 0.038, *t* = 2.03, *p* = 0.045. Our follow‐up simple slope analyses showed that, as seen in Figure 7, analytic thinking was positively associated with science beliefs among people who valued being rational strongly (+1 SD), *b* = 0.21, SE = 0.006, *t* = 3.76, *p* < 0.001, but not among those who valued being rational weakly (−1 SD), *b* = 0.04, SE = 0.05, *t* = 0.74, *p* = 0.46. Thus, across two studies, we find reliable support for our hypothesis, although the effect was small.

**FIGURE 8:**
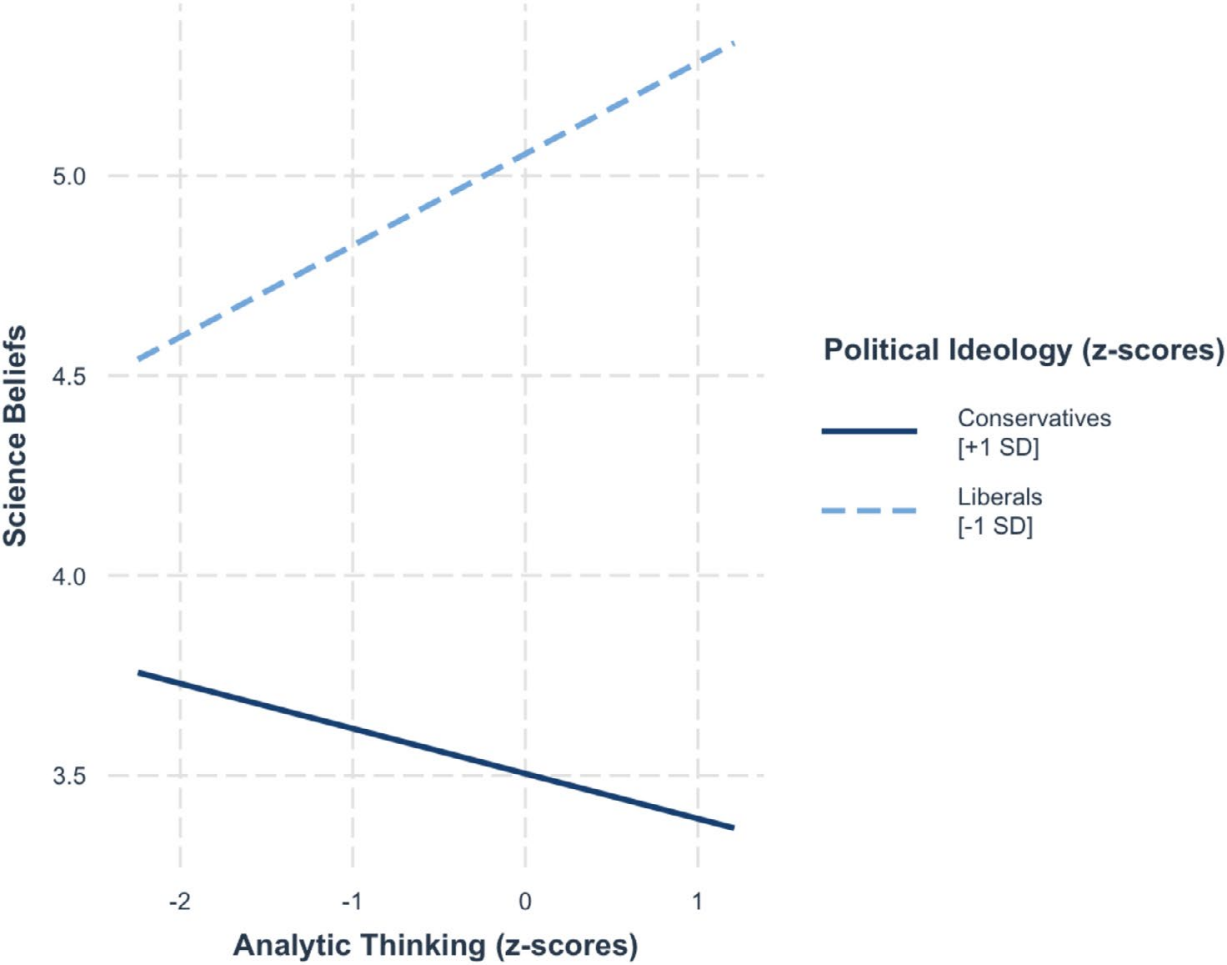
Predicted science beliefs scores as a function of analytic thinking (AT) and political ideology (Studies 1 and 2 Combined).

Moreover, when taking the data from both studies into account, these findings were not qualified by political ideology, as there was no three‐way interaction, *b* = 0.02, SE = 0.03, *t* = 0.82, *p* = 0.41. However, as in Study 2, there was a two‐way interaction between analytic thinking and political ideology, *b* = −0.17, SE = 0.03, *t* = −5.72, *p* < 0.001. As seen in Figure 8, simple slopes showed that analytic thinking was positively associated with science beliefs among liberals, *b* = 0.24, SE = 0.04, *t* = 5.47, *p* < 0.001 but negatively associated with science beliefs among conservatives, *b* = −0.11, SE = 0.04, *t* = −2.66, *p* = 0.01.

### 4.2 Skepticism Toward Alternative Medicine

**FIGURE 9:**
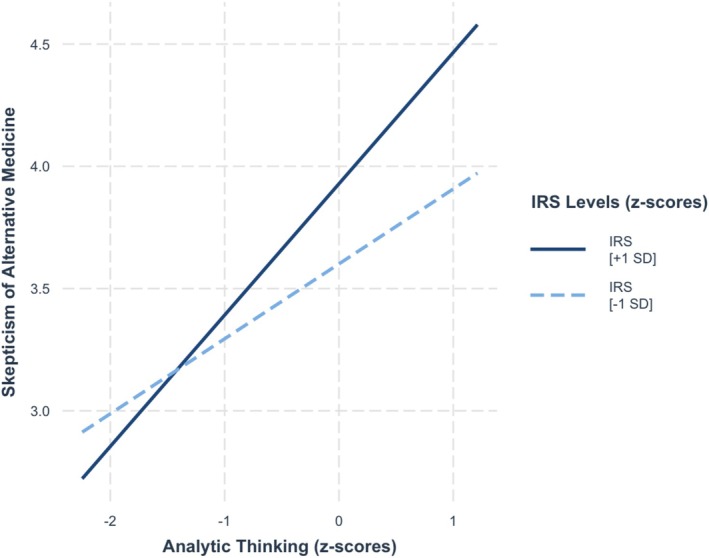
Predicted skepticism of alternative medicine scores as a function of analytic thinking (AT) and the importance of rationality (IRS) (Studies 1 and 2 Combined).

Step 1 explained a substantial amount of variance in skepticism of alternative medicine, *F*(2, 979) = 103.6, *p* < 0.001, *R* ^2^ = 0.17. Consistent with previous studies, both analytic thinking and the value given to being rational were positively associated with skepticism of alternative medicine, *b* = 0.42, SE = 0.034, *t* = 12.55, *p* < 0.001 and *b* = 0.15, SE = 0.034, *t* = 4.51, *p* < 0.001, respectively. Notably, a significant amount of additional variance was explained in Step 2, *F*(1, 978) = 9.99, *p* = 0.001, ∆*R* ^2^ = 0.01, as the value given to being rational moderated the relationship between analytic thinking and skepticism of alternative medicine, *b* = 0.11, SE = 0.033, *t* = 3.46, *p* = 0.001. Our follow‐up simple slope analyses showed that, as seen in Figure 9, analytic thinking was more strongly positively associated with skepticism of alternative medicine among people who valued being rational strongly (+ 1 SD), *b* = 0.54, SE = 0.05, *t* = 11.07, *p* < 0.001, than among those who valued being rational weakly (− 1 SD), *b* = 0.33, SE = 0.05, *t* = 7.01, *p* < 0.001. Thus, across the two studies, we found reliable support for our hypothesis for skepticism toward alternative medicine as well. Moreover, this two‐way interaction remained significant when controlling for political ideology, and it was not qualified by political ideology, as there was no three‐way interaction, *b* = 0.012, SE = 0.032, *t* = 0.037, *p* = 0.71^6^.

## 5 General Discussion

A growing literature suggests that analytic thinking promotes skepticism toward various epistemically suspect claims, and that this is especially the case when combined with strong motivation to reach epistemically rational conclusions (Adam‐Troian et al. 2019; Ståhl and van Prooijen 2018; Ståhl et al. 2024). However, previous work has focused exclusively on skepticism toward epistemically suspect beliefs, not on whether valuing rationality also promotes the positive connection between analytic thinking and pro‐science beliefs. We tried to address this limitation in the present studies by investigating how analytic thinking and epistemic rationality are associated with pro‐science beliefs. We hypothesized that the value given to being rational would moderate the relationship between analytic thinking and pro‐science beliefs. Specifically, analytic thinking would be more positively associated with pro‐science beliefs among people who value being rational strongly (rather than weakly).

In Study 1, we supported our hypotheses. Analytic thinking was associated with science beliefs and skepticism toward alternative medicine among those who value being rational strongly (rather than weakly). However, because science beliefs were highly correlated with political ideology, we conducted additional analyses to see whether political ideology qualified our results. These analyses revealed that our hypothesis was supported among conservatives, whereas this association between analytic thinking and science beliefs was not moderated by the motivation to be epistemically rational among liberals. Notably, political ideology did not qualify our results for skepticism toward alternative medicine.

In Study 2, we also received partial support for our hypothesis. As in Study 1, we found that analytic thinking was associated with skepticism toward alternative medicine among those who value being rational strongly (rather than weakly). Moreover, this pattern of results was not qualified by differences in political ideology. However, we did not replicate the focal interaction between analytic thinking and motivation to rational from Study 1 on science beliefs. Due to the somewhat inconsistent results across studies, we pooled the two datasets together to examine which patterns of results held up when taking all the data into account. In this combined dataset, we found support for our hypothesis on both measures; analytic thinking was more strongly associated with both skepticism toward alternative medicine and science beliefs among those who value being rational strongly (rather than weakly). Thus, the combination of analytic thinking and motivation to be rational is not just associated with skepticism toward unfounded beliefs as the previous research suggested (Adam‐Troian et al. 2019; Ståhl and van Prooijen 2018; Ståhl et al. 2024) but also predicts the adoption of pro‐science beliefs. In other words, the combination of analytic thinking and motivation to be rational is associated with improved truth discernment. In addition, when taking all of the data into account, none of the focal interactions were qualified by political ideology. Thus, we have no solid evidence to suggest that political ideology moderates this effect. However, it is also possible that we had insufficient statistical power to detect this three‐way interaction. Thus, future studies, with more highly powered designs, should further examine this issue.

For science beliefs, we did obtain some evidence that analytic thinking accentuates differences in belief as a function of political ideology. This pattern of results could be due to motivated reasoning or more established prior beliefs among people who score high (vs. low) on analytic thinking. By contrast, for skepticism toward alternative medicine, the results suggest that analytic thinking consistently predicts more epistemically rational beliefs. This inconsistency might stem from the politically charged nature of the beliefs assessed by our science belief scale, as compared to beliefs about alternative medicine. Indeed, our science beliefs scale was considerably more strongly associated with liberal political ideology than was the scale measuring skepticism toward alternative medicine.

### 5.1 Limitations and Suggestions for Future Research

A striking pattern in the present studies was the strong association between science beliefs and liberal political ideology. This observation is consistent with previous studies (McCright et al. 2013; Medlin et al. 2019). As a consequence, we do not know how our findings generalize to science beliefs that have not been politicized or mainly held by conservatives. In a broader sense, this association between liberal political ideology and pro‐science belief items also echoes the concerns regarding stimulus generalizability and the difficulty of finding pro‐science belief items that are supported by conservatives more than liberals. Thus, future research should attempt to develop pro‐science belief measures that are uncorrelated with political ideology and/or supported by conservatives.

An important limitation of the present studies is that they were correlational, thus preventing us from drawing conclusions about the causal effects of valuing rationality, analytic thinking, and political ideology on the endorsement of pro‐science beliefs. Despite this limitation in the present studies, experimental studies on epistemically suspect beliefs and Bullshit Receptivity (BS) receptivity have found causal evidence for the focal interaction (see Adam‐Troian et al. 2019; Ståhl et al. 2024). In light of those studies, it seems plausible that the present findings do reflect causal relationships, but this has yet to be confirmed in experimental studies.

It is also worth pointing out that the interaction effects obtained in the present studies were small (whereas the main effects of analytic thinking and valuing epistemic rationality were not). In particular, the simple slopes for skepticism toward alternative medicine were quite similar for people who strongly (and weakly) value being epistemically rational. However, analytic thinking only predicted science beliefs among people who strongly (rather than weakly) value being rational, indicating that it can play a key role in promoting more epistemically rational beliefs. Nevertheless, given the small effect sizes, it is worth considering whether other factors may be required to strengthen these relationships. For instance, the knowledge deficit account proposes that people might reject some scientific claims because they lack the basic scientific knowledge to grasp them (e.g., Bak 2001). In other words, people may reject scientifically plausible claims simply because they lack sufficient background knowledge. Indeed, Pennycook et al. (2023) found that basic knowledge of scientific facts was associated with pro‐science beliefs, even among politicized science issues. It is possible that we might have observed a stronger role of valuing rationality for the relationship between analytic thinking and pro‐science beliefs among people with basic scientific knowledge. Future studies are needed to investigate this possibility. Relatedly, future studies could examine whether teaching people about the science behind pro‐science beliefs accentuates the role of valuing rationality for the relationship between analytic thinking and pro‐science beliefs.

## 6 Conclusion

Across two studies, we supported the hypothesis that analytic thinking is more positively associated with science beliefs and skepticism toward alternative medicine among people who strongly (rather than weakly) value being rational. Thus, the combination of analytic thinking and valuing being rational is not merely associated with greater skepticism toward all truth claims. Rather, it is associated with a greater ability to discriminate between truth claims that are supported by strong evidence and claims that are not. Future studies should examine the extent to which these findings generalize to science beliefs predominantly accepted by conservatives and establish that these findings represent causal relationships.

## Author Contributions

The author takes full responsibility for this article.

## Ethics Statement

The present research has been approved by the IRB at the University of Illinois Chicago.

## Consent

All participants have given their informed consent prior to participating in these studies.

## Conflicts of Interest

The authors declare no conflicts of interest.

## Supporting information


Data S1.

## Data Availability

The data that support the findings of this study are openly available in the OSF at https://osf.io/mc2hs/?view_only=f62f091b4f8e44d2be1a0b2287b8118e.

## Endnotes

^1^
Yet, they do accept that ideology and thus values can bias cognitive processing, as ideology and prior beliefs are associated (see Tappin et al. 2020a).

^2^
Half of the participants were presented with the CRT 1–2 while the other half of the participants were presented with the pro‐scientific beliefs items first. Both groups then answered some demographic questions at the end of the survey. The order of the measures did not change the results.

^3^
The reported scale consisted of four items: global warming, big bang, evolution, and sex.

^4^
The reported scale consisted of six items: GMO, acupuncture, vaccine, homeopathy, oils, and detox.

^5^
Results remained significant with the nuclear power item included in the scale, *b* = 0.10, SE = 0.04, *t* = 2.39, *p* = 0.017.

^6^
Based on a request from an anonymous reviewer, we ran additional analyses in which we also controlled for gender, age, and level of education. The focal interaction remained significant for skepticism of alternative medicine, *b* = 0.10, SE = 0.04, *p* = 0.004, and marginally significant for science beliefs, *b* = 0.07, SE = 0.04, *p* = 0.057. For tables with the complete regression models, for the combined dataset, as well as for each study separately, see Supporting Information S1.
