# Supplementary material for: Pro‐Science Beliefs: The Role of Analytic Thinking and Epistemic Values
Source: Scand J Psychol. 2025 Apr 3;66(5):702–16. doi: 10.1111/sjop.13114 (PMC12423745; doi:10.1111/sjop.13114)
Supplement: Supplementary file 1 — Data S1. [file SJOP-66-702-s001.zip › sjop13114-sup-0001-Controlling for Demographics.docx]

Science beliefs as a function of AT and IRS controlling age, gender, and education

|  | Science Beliefs Study 1 | | | Science Beliefs Study 2 | | | Science Beliefs Study 1-2 combined | | |
| --- | --- | --- | --- | --- | --- | --- | --- | --- | --- |
| *Predictors* | *Estimates* | *CI* | *p* | *Estimates* | *CI* | *p* | *Estimates* | *CI* | *p* |
| (Intercept) | 4.06 | 3.74 – 4.39 | **<0.001** | 4.22 | 3.90 – 4.54 | **<0.001** | 4.13 | 3.90 – 4.36 | **<0.001** |
| Age | -0.04 | -0.14 – 0.06 | 0.473 | -0.19 | -0.30 – -0.08 | **0.001** | -0.11 | -0.18 – -0.03 | **0.006** |
| Gender | 0.23 | 0.03 – 0.43 | **0.026** | -0.03 | -0.24 – 0.17 | 0.758 | 0.11 | -0.04 – 0.25 | 0.151 |
| Education | 0.04 | -0.06 – 0.15 | 0.400 | 0.13 | 0.02 – 0.23 | **0.021** | 0.08 | 0.00 – 0.15 | **0.048** |
| AT | 0.13 | 0.02 – 0.23 | **0.019** | 0.11 | 0.00 – 0.22 | **0.044** | 0.12 | 0.04 – 0.19 | **0.003** |
| IRS | 0.39 | 0.29 – 0.50 | **<0.001** | 0.46 | 0.35 – 0.57 | **<0.001** | 0.42 | 0.34 – 0.49 | **<0.001** |
| AT*IRS | 0.13 | 0.03 – 0.23 | **0.013** | 0.02 | -0.08 – 0.13 | 0.673 | 0.07 | -0.00 – 0.15 | 0.057 |
| Observations | 470 | | | 512 | | | 982 | | |
| R^2^ / R^2^ adjusted | 0.138 / 0.127 | | | 0.158 / 0.148 | | | 0.134 / 0.129 | | |

Skepticism of Alternative Medicine as a function of AT and IRS controlling age, gender, and education

|  | Skepticism of Alternative Medicine Study 1 | | | Skepticism of Alternative Medicine Study 2 | | | Skepticism of Alternative Medicine Study 1-2 Combined | | |
| --- | --- | --- | --- | --- | --- | --- | --- | --- | --- |
| *Predictors* | *Estimates* | *CI* | *p* | *Estimates* | *CI* | *p* | *Estimates* | *CI* | *p* |
| (Intercept) | 4.41 | 4.15 – 4.67 | **<0.001** | 4.22 | 3.60 – 4.14 | **<0.001** | 4.07 | 3.87 – 4.27 | **<0.001** |
| Age | -0.16 | -0.24 – -0.08 | **<0.001** | -0.15 | -0.24 – -0.06 | **0.001** | -0.16 | -0.23 – -0.10 | **<0.001** |
| Gender | -0.42 | -0.58 – -0.26 | **<0.001** | -0.11 | -0.28 – 0.07 | 0.228 | -0.20 | -0.33 – -0.08 | **0.002** |
| Education | -0.02 | -0.10 – 0.06 | 0.598 | -0.002 | -0.09 – 0.09 | **0.95** | -0.02 | -0.08 – 0.05 | 0.602 |
| AT | 0.28 | 0.19 – 0.36 | **<0.001** | 0.51 | 0.43 – 0.61 | **<0.001** | 0.42 | 0.36 – 0.49 | **<0.001** |
| IRS | 0.09 | 0.00 – 0.17 | **0.041** | 0.20 | 0.11 – 0.30 | **<0.001** | 0.17 | 0.10 – 0.24 | **<0.001** |
| AT*IRS | 0.07 | -0.02 – 0.15 | 0.115 | 0.10 | 0.00 – 0.19 | **0.03** | 0.10 | 0.03 – 0.16 | **0.004** |
| Observations | 470 | | | 512 | | | 982 | | |
| R^2^ / R^2^ adjusted | 0.183 / 0.172 | | | 0.264 / 0.256 | | | 0.207 / 0.202 | | |
