# Supplementary material for: Pro‐Science Beliefs: The Role of Analytic Thinking and Epistemic Values
Source: Scand J Psychol. 2025 Apr 3;66(5):702–16. doi: 10.1111/sjop.13114 (PMC12423745; doi:10.1111/sjop.13114)
Supplement: Supplementary file 1 — Data S1. [file SJOP-66-702-s001.zip › sjop13114-sup-0002-ProScienceBeliefs_SupplementaryMaterials_Final.docx]

**Study 1: Factor analyses of science-belief items**

We conducted an exploratory factor analysis with Minimum Residuals and “oblimin” rotation. Results for our tests of dimensionality are shown in Figure S1. The scree line for our data is presented in black. The scree-test yields two factors above the scree line. Additional tests that were run as described in the nFactors package suggested a three-factor solution (but the loadings of items in this factor model were weak and/or mainly carried by one or two strongly loaded items).


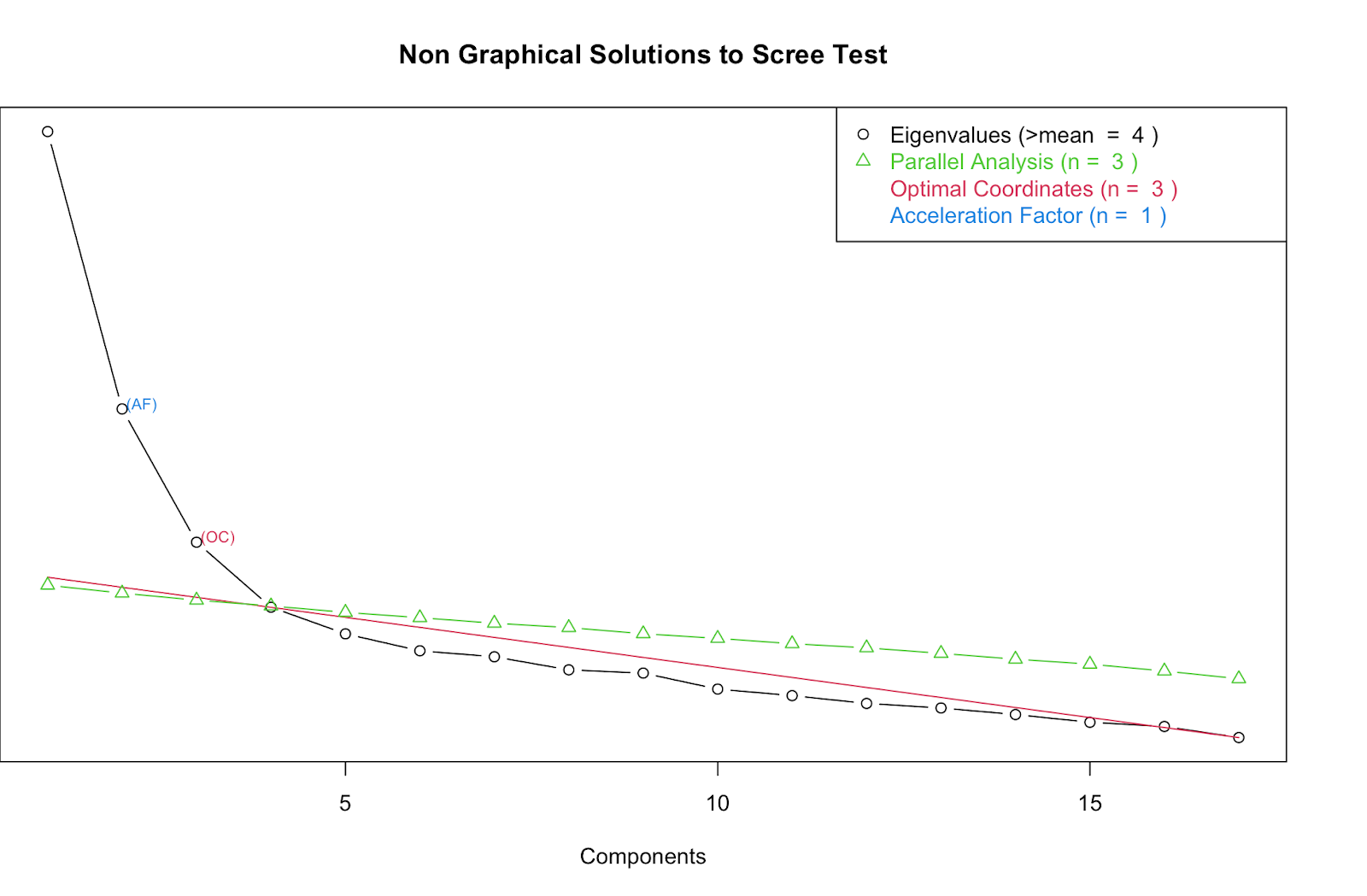


As can be seen in Table S1, one factor describes shared variation in beliefs about vaccine, nuclear power, homeopathy, GMO, acupuncture, oils, and detox (detailed descriptions of the items can be seen at the end of the SM). We refer to this factor as “Skepticism toward alternative medicine''. The second factor describes shared variation in beliefs about global warming, biological sex differences (referred to as sexr), evolution, and the big bang. We will refer to this factor as “Science Beliefs”.

The Skepticism toward alternative medicine and Science beliefs factors were positively correlated (.43). The remaining items, SAT, gtrans, gender wage gap (referred to as wage-)^^[[1]](#footnote-1)^^, stereotypes, testosterone, and IQ heritability, did not load sufficiently on either factor (< .4). As a result, these items were dropped from further analyses. The results of a confirmatory factor analysis showed that this model fit the data reasonably well, $X2$(43) = 202.35, *p* < .001, RMSEA = .088, CFI = .911, TLI =.886, BIC = 17331.10, AIC = 17331.1.

Table S1: EFA Loadings (Study 1)^^[[2]](#footnote-2)^^

Factor 1 Factor 2

globalwarm 0.02 **0.65**

wage 0.20 -**0.62**

sexr 0.09 **0.61**

evolution 0.29 **0.49**

bigbang 0.30 **0.44**

stereotypes 0.09 0.33

vaccine **0.54** 0.30

gtrans 0.08 -**0.44**

nuclearpower **0.40** -0.30

testosterone 0.15 -0.33

iqheritable 0.00 -0.06

homepath **0.74** 0.00

gmo **0.62** 0.10

acupunct **0.70** -0.16

oils **0.78** -0.01

detox **0.64** 0.07

sat 0.02 -0.30

**Study 2: Confirmatory factor analysis**

We fitted the two-factor model obtained in Study 1 to the data from Study 2, allowing the two factors to correlate. The results showed that this model fits the data reasonably well, $X2$(53) = 491.095, *p* < .001, RMSEA = .127, CFI = .828, TLI =.786. As can be seen in Table S2, the only difference was the weak loading of the nuclear power item on the skepticism of alternative medicine factor. When the nuclear power item was excluded, the results showed that this model fits the data reasonably well, $X$^2^(43) = 383.247, *p* < .001, RMSEA = .124, CFI = .86, TLI = .821.^^[[3]](#footnote-3)^^ Therefore, we opted to drop the nuclear power item from our analyses in both studies. As was the case in Study 1, the two factors were correlated positively (.41). Both factors were negatively associated with conservatism (*r* = -.27 and *r* = -.65, respectively).

Table S2: CFA Loadings

Factor 1 Factor 2

globalwarm **0.47**

sexr **0.45**

evolution **0.91**

bigbang **0.89**

vaccine **0.65**

nuclearpower 0.20

homepath **0.73**

gmo **0.67**

acupunct **0.65**

oils **0.82**

detox **0.69**

**Regression Analyses Controlling for Political Ideology**

**Study 1**

**Table S3**

Science beliefs as a function of AT, IRS, and political ideology (Study 1)

| \|  \| **Science Beliefs** \| \| \| **Science Beliefs** \| \| \| \| --- \| --- \| --- \| --- \| --- \| --- \| --- \| \| *Predictors* \| *Estimates* \| *CI* \| *p* \| *Estimates* \| *CI* \| *p* \| \| (Intercept) \| 4.41 \| 4.31 – 4.52 \| **<0.001** \| 4.43 \| 4.35 – 4.51 \| **<0.001** \| \| AT \| 0.11 \| 0.01 – 0.22 \| **0.030** \| 0.10 \| 0.02 – 0.18 \| **0.011** \| \| IRS \| 0.38 \| 0.28 – 0.49 \| **<0.001** \| 0.25 \| 0.17 – 0.33 \| **<0.001** \| \| AT * IRS \| 0.12 \| 0.02 – 0.22 \| **0.021** \| 0.08 \| 0.00 – 0.16 \| **0.042** \| \| Political Ideology \|  \|  \|  \| -0.73 \| -0.81 – -0.65 \| **<0.001** \| \| AT*Political Ideology \|  \|  \|  \| -0.11 \| -0.19 – -0.03 \| **0.007** \| \| IRS*Political Ideology \|  \|  \|  \| 0.07 \| -0.01 – 0.15 \| 0.097 \| \| AT*IRS*Political Ideology \|  \|  \|  \| 0.11 \| 0.03 – 0.18 \| **0.007** \| \| Observations \| 470 \| \| \| 470 \| \| \| \| R^2^ / R^2^ adjusted \| 0.127 / 0.121 \| \| \| 0.497 / 0.489 \| \| \| |
| --- | --- | --- | --- | --- | --- | --- | --- | --- | --- | --- | --- | --- | --- | --- | --- | --- | --- | --- | --- | --- | --- | --- | --- | --- | --- | --- | --- | --- | --- | --- | --- | --- | --- | --- | --- | --- | --- | --- | --- | --- | --- | --- | --- | --- | --- | --- | --- | --- | --- | --- | --- | --- | --- | --- | --- | --- | --- | --- | --- | --- | --- | --- | --- | --- | --- | --- | --- | --- | --- | --- | --- | --- | --- | --- | --- | --- | --- | --- | --- | --- | --- | --- | --- | --- |

**Table S4**

| Skepticism of Altenative Medicine as a function of AT, IRS, and political ideology (Study 1) |
| --- |
| \|  \| **Skepticism of Alternative Medicine** \| \| \| **Skepticism of Alternative Medicine** \| \| \| \| --- \| --- \| --- \| --- \| --- \| --- \| --- \| \| *Predictors* \| *Estimates* \| *CI* \| *p* \| *Estimates* \| *CI* \| *p* \| \| (Intercept) \| 3.82 \| 3.72 – 3.92 \| **<0.001** \| 3.81 \| 3.72 – 3.91 \| **<0.001** \| \| AT \| 0.32 \| 0.22 – 0.42 \| **<0.001** \| 0.31 \| 0.22 – 0.41 \| **<0.001** \| \| IRS \| 0.14 \| 0.04 – 0.24 \| **0.006** \| 0.07 \| -0.03 – 0.16 \| 0.154 \| \| AT*IRS \| 0.11 \| 0.02 – 0.21 \| **0.018** \| 0.10 \| 0.01 – 0.20 \| **0.024** \| \| Political Ideology \|  \|  \|  \| -0.34 \| -0.44 – -0.25 \| **<0.001** \| \| AT*Political Ideology \|  \|  \|  \| 0.02 \| -0.07 – 0.12 \| 0.648 \| \| IRS*Political Ideology \|  \|  \|  \| -0.07 \| -0.16 – 0.03 \| 0.175 \| \| AT*IRS*Political Ideology \|  \|  \|  \| 0.07 \| -0.02 – 0.16 \| 0.141 \| \| Observations \| 470 \| \| \| 470 \| \| \| \| R^2^ / R^2^ adjusted \| 0.112 / 0.106 \| \| \| 0.206 / 0.194 \| \| \| |

**Study 2**

**Science Beliefs.**

**Table S5**

Science beliefs as a function of AT, IRS, and political ideology (Study 1)

|  | **Science Beliefs** | | | **Science Beliefs** | | |
| --- | --- | --- | --- | --- | --- | --- |
| *Predictors* | *Estimates* | *CI* | *p* | *Estimates* | *CI* | *p* |
| (Intercept) | 4.17 | 4.06 – 4.28 | **<0.001** | 4.14 | 4.06 – 4.23 | **<0.001** |
| AT | 0.11 | -0.00 – 0.22 | 0.058 | 0.02 | -0.06 – 0.11 | 0.590 |
| IRS | 0.44 | 0.33 – 0.55 | **<0.001** | 0.30 | 0.21 – 0.38 | **<0.001** |
| AT*IRS | 0.04 | -0.07 – 0.15 | 0.455 | 0.01 | -0.08 – 0.09 | 0.859 |
| Political Ideology |  |  |  | -0.78 | -0.86 – -0.69 | **<0.001** |
| AT*Political Ideology |  |  |  | -0.21 | -0.30 – -0.12 | **<0.001** |
| IRS*Political Ideology |  |  |  | 0.00 | -0.08 – 0.09 | 0.967 |
| AT*IRS*Political Ideology |  |  |  | -0.03 | -0.12 – 0.07 | 0.576 |
| Observations | 512 | | | 512 | | |
| R^2^ / R^2^ adjusted | 0.130 / 0.125 | | | 0.497 / 0.490 | | |

**Skepticism Toward Alternative Medicine.**

| **Table S6**  Skepticism of Alternative Medicine as a function of AT, IRS, and political ideology (Study 2)   \|  \| Skepticism of Alternative Medicine \| \| \| Skepticism of Alternative Medicine \| \| \| \| --- \| --- \| --- \| --- \| --- \| --- \| --- \| \| *Predictors* \| *Estimates* \| *CI* \| *p* \| *Estimates* \| *CI* \| *p* \| \| (Intercept) \| 3.71 \| 3.62 – 3.81 \| **<0.001** \| 3.70 \| 3.61 – 3.78 \| **<0.001** \| \| AT \| 0.51 \| 0.42 – 0.60 \| **<0.001** \| 0.46 \| 0.37 – 0.54 \| **<0.001** \| \| IRS \| 0.19 \| 0.10 – 0.28 \| **<0.001** \| 0.13 \| 0.04 – 0.21 \| **0.005** \| \| AT*IRS \| 0.11 \| 0.02 – 0.20 \| **0.021** \| 0.09 \| 0.00 – 0.17 \| **0.047** \| \| Political Ideology \|  \|  \|  \| -0.40 \| -0.48 – -0.31 \| **<0.001** \| \| AT*Political Ideology \|  \|  \|  \| -0.03 \| -0.12 – 0.06 \| 0.471 \| \| IRS*Political Ideology \|  \|  \|  \| -0.09 \| -0.17 – -0.00 \| **0.050** \| \| AT*IRS*Political Ideology \|  \|  \|  \| -0.02 \| -0.11 – 0.07 \| 0.666 \| \| Observations \| 512 \| \| \| 512 \| \| \| \| R^2^ / R^2^ adjusted \| 0.244 / 0.240 \| \| \| 0.367 / 0.358 \| \| \| |
| --- | --- | --- | --- | --- | --- | --- | --- | --- | --- | --- | --- | --- | --- | --- | --- | --- | --- | --- | --- | --- | --- | --- | --- | --- | --- | --- | --- | --- | --- | --- | --- | --- | --- | --- | --- | --- | --- | --- | --- | --- | --- | --- | --- | --- | --- | --- | --- | --- | --- | --- | --- | --- | --- | --- | --- | --- | --- | --- | --- | --- | --- | --- | --- | --- | --- | --- | --- | --- | --- | --- | --- | --- | --- | --- | --- | --- | --- | --- | --- | --- | --- | --- | --- | --- |

**Studies 1-2 Combined**

**Science Beliefs.**

| **Table S7**  Science beliefs as a function of AT, IRS, and political ideology (Study 1 and 2)   \|  \| **Science Beliefs** \| \| \| **Science Beliefs** \| \| \| \| --- \| --- \| --- \| --- \| --- \| --- \| --- \| \| *Predictors* \| *Estimates* \| *CI* \| *p* \| *Estimates* \| *CI* \| *p* \| \| (Intercept) \| 4.29 \| 4.21 – 4.36 \| **<0.001** \| 4.28 \| 4.22 – 4.34 \| **<0.001** \| \| AT \| 0.11 \| 0.03 – 0.19 \| **0.004** \| 0.06 \| -0.00 – 0.12 \| 0.051 \| \| IRS \| 0.41 \| 0.33 – 0.49 \| **<0.001** \| 0.27 \| 0.21 – 0.33 \| **<0.001** \| \| AT*IRS \| 0.08 \| 0.00 – 0.15 \| **0.045** \| 0.04 \| -0.02 – 0.10 \| 0.173 \| \| Political Ideology \|  \|  \|  \| -0.78 \| -0.83 – -0.72 \| **<0.001** \| \| AT*Political Ideology \|  \|  \|  \| -0.17 \| -0.23 – -0.11 \| **<0.001** \| \| IRS*Political Ideology \|  \|  \|  \| 0.04 \| -0.01 – 0.10 \| 0.141 \| \| AT*IRS*Political Ideology \|  \|  \|  \| 0.02 \| -0.03 – 0.08 \| 0.410 \| \| Observations \| 982 \| \| \| 982 \| \| \| \| R^2^ / R^2^ adjusted \| 0.123 / 0.120 \| \| \| 0.496 / 0.492 \| \| \| |
| --- | --- | --- | --- | --- | --- | --- | --- | --- | --- | --- | --- | --- | --- | --- | --- | --- | --- | --- | --- | --- | --- | --- | --- | --- | --- | --- | --- | --- | --- | --- | --- | --- | --- | --- | --- | --- | --- | --- | --- | --- | --- | --- | --- | --- | --- | --- | --- | --- | --- | --- | --- | --- | --- | --- | --- | --- | --- | --- | --- | --- | --- | --- | --- | --- | --- | --- | --- | --- | --- | --- | --- | --- | --- | --- | --- | --- | --- | --- | --- | --- | --- | --- | --- | --- |

**Skepticism Toward Alternative Medicine.**

| **Table S8**  Skepticism of Alternative Medicine as a function of AT, IRS, and political ideology (Study 1 and 2)   \|  \| Skepticism of Alternative Medicine \| \| \| Skepticism of Alternative Medicine \| \| \| \| --- \| --- \| --- \| --- \| --- \| --- \| --- \| \| *Predictors* \| *Estimates* \| *CI* \| *p* \| *Estimates* \| *CI* \| *p* \| \| (Intercept) \| 3.76 \| 3.70 – 3.83 \| **<0.001** \| 3.76 \| 3.69 – 3.82 \| **<0.001** \| \| AT \| 0.42 \| 0.35 – 0.49 \| **<0.001** \| 0.39 \| 0.32 – 0.45 \| **<0.001** \| \| IRS \| 0.16 \| 0.10 – 0.23 \| **<0.001** \| 0.10 \| 0.03 – 0.16 \| **0.003** \| \| AT*IRS \| 0.12 \| 0.05 – 0.18 \| **0.001** \| 0.10 \| 0.04 – 0.16 \| **0.002** \| \| Political Ideology \|  \|  \|  \| -0.38 \| -0.45 – -0.32 \| **<0.001** \| \| AT*Political Ideology \|  \|  \|  \| 0.00 \| -0.06 – 0.07 \| 0.940 \| \| IRS*Political Ideology \|  \|  \|  \| -0.06 \| -0.13 – -0.00 \| **0.042** \| \| AT*IRS*Political Ideology \|  \|  \|  \| 0.01 \| -0.05 – 0.07 \| 0.714 \| \| Observations \| 982 \| \| \| 982 \| \| \| \| R^2^ / R^2^ adjusted \| 0.176 / 0.174 \| \| \| 0.287 / 0.282 \| \| \|  \| Skepticism of Alternative Medicine as a function of AT, IRS, and political ideology (Study 1 and 2) \| \| --- \| |
| --- | --- | --- | --- | --- | --- | --- | --- | --- | --- | --- | --- | --- | --- | --- | --- | --- | --- | --- | --- | --- | --- | --- | --- | --- | --- | --- | --- | --- | --- | --- | --- | --- | --- | --- | --- | --- | --- | --- | --- | --- | --- | --- | --- | --- | --- | --- | --- | --- | --- | --- | --- | --- | --- | --- | --- | --- | --- | --- | --- | --- | --- | --- | --- | --- | --- | --- | --- | --- | --- | --- | --- | --- | --- | --- | --- | --- | --- | --- | --- | --- | --- | --- | --- | --- | --- |

**Regression Results Using Each Removed Science Beliefs Item**

*Regression results using Nuclear power as the criterion (Study 1 and 2 Combined)*

| Predictor | *b* | *b*  95% CI [LL, UL] | *sr^2^* | *sr^2^*  95% CI [LL, UL] | Fit |
| --- | --- | --- | --- | --- | --- |
| (Intercept) | 4.07** | [3.97, 4.16] |  |  |  |
| AT | 0.23** | [0.14, 0.33] | .02 | [.00, .04] |  |
| IRS | 0.14** | [0.04, 0.23] | .01 | [-.00, .02] |  |
| AT:IRS | 0.07 | [-0.03, 0.16] | .00 | [-.00, .01] |  |
|  |  |  |  |  | *R^2^*   = .038** |
|  |  |  |  |  | 95% CI[.02,.06] |
|  |  |  |  |  |  |

*Regression results using Stereotypes as the criterion (Study 1 and 2 Combined)*

| Predictor | *b* | *b*  95% CI [LL, UL] | *sr^2^* | *sr^2^*  95% CI [LL, UL] | Fit |
| --- | --- | --- | --- | --- | --- |
| (Intercept) | 5.09** | [5.01, 5.17] |  |  |  |
| AT | 0.29** | [0.22, 0.37] | .05 | [.03, .08] |  |
| IRS | 0.19** | [0.12, 0.27] | .02 | [.00, .04] |  |
| AT:IRS | -0.01 | [-0.08, 0.07] | .00 | [-.00, .00] |  |
|  |  |  |  |  | *R^2^*   = .091** |
|  |  |  |  |  | 95% CI[.06,.12] |
|  |  |  |  |  |  |

*Regression results using Gtrans as the criterion (Study 1 and 2 Combined)*

| Predictor | *b* | *b*  95% CI [LL, UL] | *sr^2^* | *sr^2^*  95% CI [LL, UL] | Fit |
| --- | --- | --- | --- | --- | --- |
| (Intercept) | 3.89** | [3.79, 3.98] |  |  |  |
| AT | -0.10* | [-0.19, -0.00] | .00 | [-.00, .01] |  |
| IRS | -0.01 | [-0.10, 0.09] | .00 | [-.00, .00] |  |
| AT:IRS | -0.06 | [-0.15, 0.04] | .00 | [-.00, .01] |  |
|  |  |  |  |  | *R^2^*   = .005 |
|  |  |  |  |  | 95% CI[.00,.02] |
|  |  |  |  |  |  |

This is the only place where AT predicts less endorsement of a science belief.

*Regression results using Wage as the criterion (Study 1 and 2 Combined)*

| Predictor | *b* | *b*  95% CI [LL, UL] | *sr^2^* | *sr^2^*  95% CI [LL, UL] | Fit |
| --- | --- | --- | --- | --- | --- |
| (Intercept) | 3.54** | [3.44, 3.65] |  |  |  |
| AT | 0.21** | [0.10, 0.32] | .01 | [-.00, .03] |  |
| IRS | -0.13* | [-0.24, -0.02] | .01 | [-.00, .01] |  |
| AT:IRS | 0.05 | [-0.06, 0.15] | .00 | [-.00, .00] |  |
|  |  |  |  |  | *R^2^*   = .018** |
|  |  |  |  |  | 95% CI[.00,.04] |
|  |  |  |  |  |  |

*Regression results using Testosterone as the criterion (Study 1 and 2 Combined)*

| Predictor | *b* | *b*  95% CI [LL, UL] | *sr^2^* | *sr^2^*  95% CI [LL, UL] | Fit |
| --- | --- | --- | --- | --- | --- |
| (Intercept) | 4.83** | [4.76, 4.90] |  |  |  |
| AT | 0.05 | [-0.02, 0.13] | .00 | [-.00, .01] |  |
| IRS | 0.18** | [0.11, 0.25] | .02 | [.00, .04] |  |
| AT:IRS | -0.01 | [-0.08, 0.06] | .00 | [-.00, .00] |  |
|  |  |  |  |  | *R^2^*   = .030** |
|  |  |  |  |  | 95% CI[.01,.05] |
|  |  |  |  |  |  |

*Regression results using IQ heritability as the criterion (Study 1 and 2 Combined)*

| Predictor | *b* | *b*  95% CI [LL, UL] | *sr^2^* | *sr^2^*  95% CI [LL, UL] | Fit |
| --- | --- | --- | --- | --- | --- |
| (Intercept) | 4.34** | [4.26, 4.42] |  |  |  |
| AT | -0.01 | [-0.09, 0.07] | .00 | [-.00, .00] |  |
| IRS | 0.17** | [0.09, 0.25] | .02 | [.00, .03] |  |
| AT:IRS | -0.08 | [-0.16, 0.00] | .00 | [-.00, .01] |  |
|  |  |  |  |  | *R^2^*   = .023** |
|  |  |  |  |  | 95% CI[.01,.04] |
|  |  |  |  |  |  |

*Regression results using SAT as the criterion (Study 1 and 2 Combined)*

| Predictor | *b* | *b*  95% CI [LL, UL] | *sr^2^* | *sr^2^*  95% CI [LL, UL] | Fit |
| --- | --- | --- | --- | --- | --- |
| (Intercept) | 2.99** | [2.90, 3.08] |  |  |  |
| AT | 0.03 | [-0.06, 0.12] | .00 | [-.00, .00] |  |
| IRS | -0.09* | [-0.18, -0.00] | .00 | [-.00, .01] |  |
| AT:IRS | -0.03 | [-0.12, 0.05] | .00 | [-.00, .00] |  |
|  |  |  |  |  | *R^2^*   = .005 |
|  |  |  |  |  | 95% CI[.00,.01] |
|  |  |  |  |  |  |

**Regression Results with the Original Scale**

*Results of Regression Pro-Science Beliefs (PSB) as DV. (Study 1). AT = Analytic Thinking. IRS = The Importance of Rationality Scale.*

|  | **Pro-Science Beliefs (PSB)** | | | **PSB** | | |
| --- | --- | --- | --- | --- | --- | --- |
| *Predictors* | *Estimates* | *CI* | *p* | *Estimates* | *CI* | *p* |
| (Intercept) | 4.06 | 4.01 – 4.11 | **<0.001** | 4.06 | 4.01 – 4.11 | **<0.001** |
| AT | 0.17 | 0.12 – 0.22 | **<0.001** | 0.17 | 0.12 – 0.22 | **<0.001** |
| IRS | 0.16 | 0.11 – 0.21 | **<0.001** | 0.13 | 0.08 – 0.18 | **<0.001** |
| AT*IRS | 0.06 | 0.01 – 0.11 | **0.020** | 0.05 | 0.01 – 0.10 | **0.030** |
| Political Ideology |  |  |  | -0.18 | -0.23 – -0.13 | **<0.001** |
| AT*Political Ideology |  |  |  | -0.00 | -0.05 – 0.05 | 0.872 |
| IRS*Political Ideology |  |  |  | -0.00 | -0.05 – 0.05 | 0.907 |
| AT*IRS*Political Ideology |  |  |  | 0.05 | 0.00 – 0.10 | **0.047** |
| Observations | 470 | | | 470 | | |
| R^2^ / R^2^ adjusted | 0.171 / 0.165 | | | 0.261 / 0.249 | | |

*Results of Regression Liberal Pro-Science Beliefs (PSB) as DV. (Study 1). AT = Analytic Thinking. IRS = The Importance of Rationality Scale.*

|  | **Liberal PSB** | | | **Liberal PSB** | | |
| --- | --- | --- | --- | --- | --- | --- |
| *Predictors* | *Estimates* | *CI* | *p* | *Estimates* | *CI* | *p* |
| (Intercept) | 3.95 | 3.90 – 4.01 | **<0.001** | 3.95 | 3.89 – 4.01 | **<0.001** |
| AT | 0.19 | 0.13 – 0.24 | **<0.001** | 0.19 | 0.13 – 0.25 | **<0.001** |
| IRS | 0.09 | 0.04 – 0.15 | **0.001** | 0.09 | 0.03 – 0.15 | **0.003** |
| AT*IRS | 0.04 | -0.01 – 0.10 | 0.134 | 0.04 | -0.01 – 0.10 | 0.108 |
| Political Ideology |  |  |  | -0.02 | -0.07 – 0.04 | 0.586 |
| AT*Political Ideology |  |  |  | 0.03 | -0.03 – 0.08 | 0.321 |
| IRS*Political Ideology |  |  |  | -0.02 | -0.08 – 0.03 | 0.394 |
| AT*IRS*Political Ideology |  |  |  | 0.03 | -0.02 – 0.09 | 0.270 |
| Observations | 470 | | | 470 | | |
| R^2^ / R^2^ adjusted | 0.118 / 0.113 | | | 0.124 / 0.110 | | |

*Results of Regression Conservative Pro-Science Beliefs (PSB) as DV. (Study 1). AT = Analytic Thinking. IRS = The Importance of Rationality Scale.*

|  | **Conservative PSB** | | | | **Conservative PSB** | |
| --- | --- | --- | --- | --- | --- | --- |
| *Predictors* | *Estimates* | *CI* | *p* | *Estimates* | *CI* | *p* |
| (Intercept) | 4.41 | 4.31 – 4.52 | **<0.001** | 4.43 | 4.35 – 4.51 | **<0.001** |
| AT | 0.11 | 0.01 – 0.22 | **0.030** | 0.10 | 0.02 – 0.18 | **0.011** |
| IRS | 0.38 | 0.28 – 0.49 | **<0.001** | 0.25 | 0.17 – 0.33 | **<0.001** |
| AT*IRS | 0.12 | 0.02 – 0.22 | **0.021** | 0.08 | 0.00 – 0.16 | **0.042** |
| Political Ideology |  |  |  | -0.73 | -0.81 – -0.65 | **<0.001** |
| AT*Political Ideology |  |  |  | -0.11 | -0.19 – -0.03 | **0.007** |
| IRS*Political Ideology |  |  |  | 0.07 | -0.01 – 0.15 | 0.097 |
| AT*IRS*Political Ideology |  |  |  | 0.11 | 0.03 – 0.18 | **0.007** |
| Observations | 470 | | | 470 | | |
| R^2^ / R^2^ adjusted | 0.127 / 0.121 | | | 0.497 / 0.489 | | |

*Results of Regression Pro-Science Beliefs (PSB) as DV. (Study 2). AT = Analytic Thinking. IRS = The Importance of Rationality Scale.*

|  | **PSB** | | | **PSB** | | |
| --- | --- | --- | --- | --- | --- | --- |
| *Predictors* | *Estimates* | *CI* | *p* | *Estimates* | *CI* | *p* |
| (Intercept) | 4.00 | 3.95 – 4.05 | **<0.001** | 3.99 | 3.94 – 4.04 | **<0.001** |
| AT | 0.26 | 0.21 – 0.31 | **<0.001** | 0.23 | 0.18 – 0.28 | **<0.001** |
| IRS | 0.20 | 0.15 – 0.25 | **<0.001** | 0.16 | 0.11 – 0.21 | **<0.001** |
| AT*IRS | 0.05 | -0.00 – 0.10 | 0.065 | 0.04 | -0.01 – 0.09 | 0.098 |
| Political Ideology |  |  |  | -0.23 | -0.28 – -0.18 | **<0.001** |
| AT*Political Ideology |  |  |  | -0.01 | -0.06 – 0.04 | 0.809 |
| IRS*Political Ideology |  |  |  | -0.03 | -0.08 – 0.02 | 0.216 |
| AT*IRS*Analytic Thinking |  |  |  | -0.01 | -0.06 – 0.04 | 0.764 |
| Observations | 512 | | | 512 | | |
| R^2^ / R^2^ adjusted | 0.284 / 0.280 | | | 0.403 / 0.395 | | |

*Results of Regression Liberal Pro-Science Beliefs (PSB) as DV. (Study 2). AT = Analytic Thinking. IRS = The Importance of Rationality Scale.*

|  | **Liberal PSB** | | | **Liberal PSB** | | |
| --- | --- | --- | --- | --- | --- | --- |
| *Predictors* | *Estimates* | *CI* | *p* | *Estimates* | *CI* | *p* |
| (Intercept) | 4.17 | 4.06 – 4.28 | **<0.001** | 4.14 | 4.06 – 4.23 | **<0.001** |
| AT | 0.11 | -0.00 – 0.22 | 0.058 | 0.02 | -0.06 – 0.11 | 0.590 |
| IRS | 0.44 | 0.33 – 0.55 | **<0.001** | 0.30 | 0.21 – 0.38 | **<0.001** |
| AT*IRS | 0.04 | -0.07 – 0.15 | 0.455 | 0.01 | -0.08 – 0.09 | 0.859 |
| Political Ideology |  |  |  | -0.78 | -0.86 – -0.69 | **<0.001** |
| AT*Political Ideology |  |  |  | -0.21 | -0.30 – -0.12 | **<0.001** |
| IRS*Political Ideology |  |  |  | 0.00 | -0.08 – 0.09 | 0.967 |
| AT*IRS*Political Ideology |  |  |  | -0.03 | -0.12 – 0.07 | 0.576 |
| Observations | 512 | | | 512 | | |
| R^2^ / R^2^ adjusted | 0.130 / 0.125 | | | 0.497 / 0.490 | | |

*Results of Regression Conservative Pro-Science Beliefs (PSB) as DV. (Study 2). AT = Analytic Thinking. IRS = The Importance of Rationality Scale.*

|  | **Conservative PSB** | | | **Conservative PSB** | | |
| --- | --- | --- | --- | --- | --- | --- |
| *Predictors* | *Estimates* | *CI* | *p* | *Estimates* | *CI* | *p* |
| (Intercept) | 3.94 | 3.89 – 4.00 | **<0.001** | 3.95 | 3.89 – 4.00 | **<0.001** |
| AT | 0.31 | 0.26 – 0.36 | **<0.001** | 0.29 | 0.24 – 0.34 | **<0.001** |
| IRS | 0.13 | 0.08 – 0.18 | **<0.001** | 0.12 | 0.07 – 0.18 | **<0.001** |
| AT*IRS | 0.05 | -0.00 – 0.10 | 0.058 | 0.05 | -0.00 – 0.10 | 0.060 |
| Political Ideology |  |  |  | -0.06 | -0.12 – -0.01 | **0.017** |
| AT*Political Ideology |  |  |  | 0.06 | 0.00 – 0.11 | **0.045** |
| IRS*Political Ideology |  |  |  | -0.04 | -0.09 – 0.01 | 0.134 |
| AT*IRS*Political Ideology |  |  |  | -0.00 | -0.06 – 0.05 | 0.938 |
| Observations | 512 | | | 512 | | |
| R^2^ / R^2^ adjusted | 0.275 / 0.271 | | | 0.292 / 0.282 | | |

**Full item coding for Pro-science Beliefs Items**

**Wage**. The gender wage gap is entirely due to sexism and nothing else. (rev)

**Gtrans**. After transitioning genders, some people change their mind and then re-transition to their original gender.

**Testosterone**. Having higher testosterone generally provides an advantage in athletic performance.

**IQheritable**. Human intelligence is moderately heritable (that is, intelligence is partly determined by genetics).

**SAT**. There are no consistent differences between men and women (on average) in terms of math scores on the SAT.  (rev)

**Nuclearpower**. Nuclear power is a relatively safe and viable source of energy.

**Globalwarm**. Global warming is at least partly caused by human activity and is a serious problem for the environment.

**Bigbang**. The big bang theory is, generally speaking, the best explanation we have so far for the origin of our universe.

**Evolution**. Evolution is the best explanation so far for our origins.

**Sexr**. Among both humans and other animals, there are only two biological sexes (male and female) with no ambiguity. (rev)

**GMO**. Genetically modified foods are hazardous to human health. (rev)

**Acupunct**. There is clear evidence that acupuncture is effective at treating illnesses and medical conditions. (rev)

**Oils**. There is good evidence that essential oils play a causal role in improving people’s health. (rev)

**Homeopath**. Homeopathic medicine is an effective means of treating some illnesses and diseases. (rev)

**Detox**. It is possible (and advisable) to "detox" the body from chemicals. (rev)

**Vaccine**. Vaccines can cause autism in children. (rev)

**Stereotypes**. Stereotypes are always entirely accurate. (rev)

1. Higher scores at the gtrans and wage items indicated a greater pro-science position, yet both items negatively loaded on holding science beliefs. Thus, we removed these items from further analysis. [↑](#footnote-ref-1)
2. EFA Model with factor number fixed 2: X^2^(103) = 565.12, *p* < .001, RMSEA = .098, AIC = 26978.27, TLI =.725, BIC = -68.62. [↑](#footnote-ref-2)
3. The original model as a CFA with liberal and conservative factor loadings, X^2^(118) = 863.247, *p* < .001, RMSEA = .111, CFI = .741, TLI = .701. [↑](#footnote-ref-3)
